# Emerging Role of 2D Materials in Photovoltaics: Efficiency Enhancement and Future Perspectives

**DOI:** 10.1007/s40820-025-01869-z

**Published:** 2025-08-18

**Authors:** Ghulam Dastgeer, Muhammad Wajid Zulfiqar, Sobia Nisar, Rimsha Zulfiqar, Muhammad Imran, Swagata Panchanan, Subhajit Dutta, Kamran Akbar, Alberto Vomiero, Zhiming Wang

**Affiliations:** 1 Department of Physics and Astronomy, Sejong University, Seoul, 05006 Republic of Korea; 2 Department of Optical Engineering, Sejong University, Seoul, 05006 Republic of Korea; 3 Department of Electrical Engineering, Sejong University, Seoul, 05006 Republic of Korea; 4 Department of Convergence Engineering for Intelligent Drone, Sejong University, Seoul, 05006 Republic of Korea; 5 Department of Chemistry, Riphah International University, Faisalabad, 44000, Pakistan; 6 Department of Chemistry, Faculty of Science, King Khalid University, P.O. Box 960, 61421 Abha, Saudi Arabia; 7 Department of Energy and Chemical Engineering, Ulsan National Institute of Science and Technology (UNIST), Ulsan, 44919 Republic of Korea; 8 Advanced Materials Science and Engineering, Sungkyunkwan University, Suwon, 16419 Republic of Korea; 9 Shimmer Center, Tianfu Jiangxi Laboratory, Chengdu, 641419 People’s Republic of China; 10 Department of Molecular Sciences and Nanosystems, Ca’ Foscari University of Venice, Via Torino 155, 30172 Venice, Italy; 11 Department of Engineering Science and Mathematics, Materials Science Division, Luleå University of Technology, 97187 Luleå, Sweden; 12 School of Physics, University of Electronic Science and Technology of China, Chengdu, 611731 People’s Republic of China

**Keywords:** 2D materials, Photovoltaics, Interface engineering, Work function tuning, Energy harvesting

## Abstract

The growing global energy demand and worsening climate change highlight the urgent need for clean, efficient and sustainable energy solutions. Among emerging technologies, atomically thin two-dimensional (2D) materials offer unique advantages in photovoltaics due to their tunable optoelectronic properties, high surface area and efficient charge transport capabilities. This review explores recent progress in photovoltaics incorporating 2D materials, focusing on their application as hole and electron transport layers to optimize bandgap alignment, enhance carrier mobility and improve chemical stability. A comprehensive analysis is presented on perovskite solar cells utilizing 2D materials, with a particular focus on strategies to enhance crystallization, passivate defects and improve overall cell efficiency. Additionally, the application of 2D materials in organic solar cells is examined, particularly for reducing recombination losses and enhancing charge extraction through work function modification. Their impact on dye-sensitized solar cells, including catalytic activity and counter electrode performance, is also explored. Finally, the review outlines key challenges, material limitations and performance metrics, offering insight into the future development of next-generation photovoltaic devices encouraged by 2D materials.

## Highlights

- A novel strategy employs 2D materials to construct cascaded band alignment, enabling efficient charge transport and reducing energy loss.
- An innovative approach utilizes donor–acceptor blends; active layer morphology and interfacial engineering minimize charge recombination to enable high performance and long-term device stability.
- This review uniquely consolidates the roles of 2D materials as electron transport layers and hole transport layers across three major classes of solar cells: perovskite, organic and dye-sensitized solar cells.

## Graphical abstract

**Figure:**
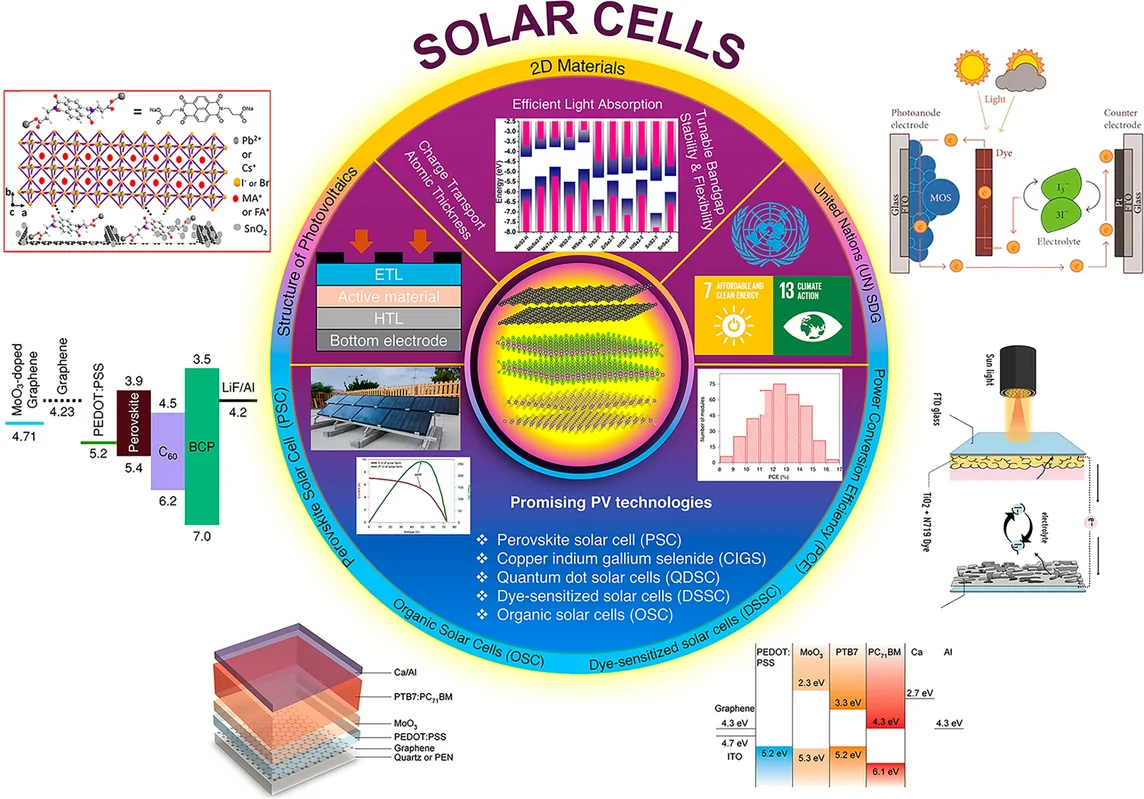

## Introduction

Amid the escalating challenges of climate change and the greenhouse effect, largely due to the continued reliance on fossil fuels, the shift toward renewable energy has emerged as a critical necessity for a sustainable future [1, 2]. The increasing demand for energy puts a strain on conventional sources, highlighting the even more importance of renewable energy coupled with environmental concerns is driving the search for cleaner and more efficient energy sources. Renewable energy offers a promising alternative to traditional fossil fuels and is gaining significant research interest [3, 4]. Unlike fossil fuels, sunlight is a constantly refillable resource, readily available in most locations and produces no harmful emissions during operation offering an inexhaustible supply of clean energy. Photovoltaic (PV) technology captures this energy and directly converts sunlight into electricity. These qualities make solar power a high-quality and sustainable choice for the future. PV technology is a cornerstone of future renewable energy production. It offers several advantages that include abundant energy resources, minimal environmental impact and long-term sustainability. PV materials convert the sun inexhaustible energy into usable forms. The development of solar cells boasts a rich history spanning over half a century [5, 6]. Over the years, the technology has progressed through silicon-based cells [7, 8], thin-film organic cells [9], including CdTe, GaAs and InP-based varieties, and the new generation of novel concept solar cells [10, 11]. Currently, silicon-based cells dominate the PV market, other promising technologies are emerging rapidly. These include perovskite solar cells (PSCs) [12, 13], copper indium gallium selenide (CIGS) solar cells [14, 15], quantum dot solar cells (QDSCs) [16, 17], dye-sensitized solar cells (DSSCs) [18, 19] and organic solar cells (OSCs) [20, 21]. Two-dimensional (2D) materials hold immense promises for revolutionizing solar cell technology. Their unique atomic structure, arranged in ultrathin layers, offers remarkable properties that make them ideal candidates for next-generation solar cells [22, 23]. The main advantage of 2D materials lies in their tunable bandgaps which represent the minimum energy difference between the material valence and its conduction band. This energy difference dictates a material's ability to absorb light and generate electrons. In 2D materials, the bandgap can be strategically modified, ranging from near-metallic behavior in graphene to semiconducting properties in black phosphorus (BP) for efficient light absorption and insulating behavior in hexagonal-boron nitride (h-BN) that is useful for controlling charge transport [24]. This remarkable control over bandgaps allows for the creation of sophisticated layered heterostructures. By carefully stacking different 2D materials, we can design solar cells with precisely tailored light absorption and charge transport properties which would result in optimizing energy conversion efficiency [25]. Recently, perovskite films are class of materials that have attracted significant research interest due to their high stability and remarkable efficiency. The quality and orientation of perovskite crystal significantly impact their performance and modifying perovskite composition can improve charge carrier mobility. Defects in the perovskite lattice can act as charge recombination center which reduce the overall cell efficiency. High-quality perovskite crystals with minimal defects and using charge selective layers can help to separate electrons and holes for reduce recombination [26, 27]. OSC solar cells offer promising potential for lightweight, low-cost cost and flexible photovoltaic devices. Trap states within the organic material act as recombination center and reduce the overall cell performance. The morphology of donor–accepter can be used for enhancing charge transport and ensuring optimal phase separation between acceptor and donor material is crucial for efficient transport [28, 29]. Counter electrodes (CE) in DSSCs facilitate the reduction of the oxidized redox mediator. 2D materials such as transition metal dichalcogenides (TMDCs), graphene and BP exhibit excellent electrical conductivity for efficient electron transfer and surface area for enhancing catalytic activity of CE. The surface area and high conductivity of these materials enhance electrocatalytic activity, which leads to faster redox mediator regeneration and higher overall cell efficiency [30, 31].

### Emerging Trends in 2D Material Use in Photovoltaics

Over the past five years, a growing body of work has demonstrated that atomically thin materials can dramatically improve the performance of several photovoltaic platforms. In perovskite solar cells (PSCs), n-type MoS_2_, WS_2_ and SnS_2_ nanosheets have been incorporated as electron transport layers (ETLs), lowering the interfacial recombination current and pushing certified power conversion efficiency (PCE) beyond 25% [32]. p-type WSe_2_ and doped graphene derivatives, when used as hole transport layers (HTLs), raise the work function at the perovskite interface and improve fill factors to > 85% [33]. Ultrathin Ti_3_C_2_T_x_ MXene and h-BN “active-buffer layers (ABLs)” further passivate surface traps and suppress ion migration, adding > 500 mV to open-circuit voltage in some inverted devices [34]. In organic solar cells (OSCs), graphene oxide and BP nanosheets embedded at the donor–acceptor interface enhance exciton dissociation and yield ∼ 20% PCEs in flexible modules [35]. For DSSCs, edge-enriched MoS_2_/RGO composites now serve as low-cost counter electrodes (CEs) with catalytic activities rivaling Pt while retaining long-term chemical stability in iodide and cobalt electrolytes [36].

### Challenges and Future Directions

Notwithstanding these advances, several obstacles still hinder the large-scale deployment of 2D-enabled PV technologies. (1) **Scalability**: continuous, wafer-scale growth or transfer of defect-free 2D films at temperatures compatible with flexible substrates remains difficult; solution-processed inks often suffer from poor flake alignment and residual surfactants. (2) **Interface stability**: chemical incompatibilities between 2D layers and hybrid perovskites can lead to ion exchange, phase segregation, or undesirable interfacial dipoles under light, heat, or moisture stress. (3) **Device longevity**: while laboratory cells may exceed 1,000 h under standard tests, the long-term (> 10,000 h) behavior of 2D/3D interfaces, especially under simultaneous thermal, electrical and mechanical cycling, has not been systematically benchmarked [37].

To translate laboratory success into bankable products, future research should prioritize (1) **scalable synthesis/printing** of orientation-controlled 2D layers via low-temperature CVD, slot-die coating, or roll-to-roll (R2R) blade coating; (2) **multifunctional heterostructures** that couple defect passivation, moisture blocking and graded band alignment in a single laminated stack; (3) **in situ and operando characterization** to track ion migration, chemical reactions and stress evolution at buried 2D/3D junctions; and (4) **data-driven screening** of 2D materials and surface chemistries using machine learning models linked to high-throughput experiments. A realistic roadmap targets > 28% PCE, < 10% performance loss after 10,000 h of damp-heat testing and fully solution-processed module fabrication by 2030 for PSCs, with analogous stability and cost metrics for OSCs and DSSCs. Achieving these milestones will position 2D-enabled photovoltaics as a competitive technology for terawatt-scale, low-carbon energy deployment [38].

### Study Gaps and Objectives

Despite significant advancements in photovoltaics, certain challenges continue to limit the full potential of these technologies. Addressing these gaps is essential for enhancing the stability and performance of solar devices, and exploring these techniques further will be critical for future applications. For 2D materials, their atomic-scale thickness limits light absorption, and sharp edges make these materials more susceptible to defects, while scalability issues constrain their commercial viability. Research into overcoming these limitations can significantly broaden the 2D materials applications in future solar devices [39, 40]. In perovskite-based photovoltaics, a major challenge lies in optimizing the interface between the charge transport layer and perovskite layer to improve charge collection and extraction. Effective interface passivation is also essential to reduce trap states and defects, which would otherwise act as recombination centers, leading to charge carrier losses. Optimization of perovskite composition and morphology will be key for enhanced device performance, and continued exploration in these areas can enable more reliable and efficient future applications [41]. In organic photovoltaics, optimizing donor–acceptor blends is necessary to reduce recombination losses, while refining the morphology of donor–acceptor composites with active layer is crucial for high efficiency. Additionally, reducing the work function of the electron transport layer (ETL) can facilitate better electron transfer from the active layer [42]. For DSSC, enhancing long-term stability and durability of both the dye and electrolyte components is crucial for extending the lifespan and performance of cell. Addressing electrolyte leakage is also critical to prevent potential hazards. Moreover, the development of efficient, cost-effective counter electrodes as alternatives to platinum (Pt) is essential for the commercial scalability of DSSCs [43].

**Fig. 1:**
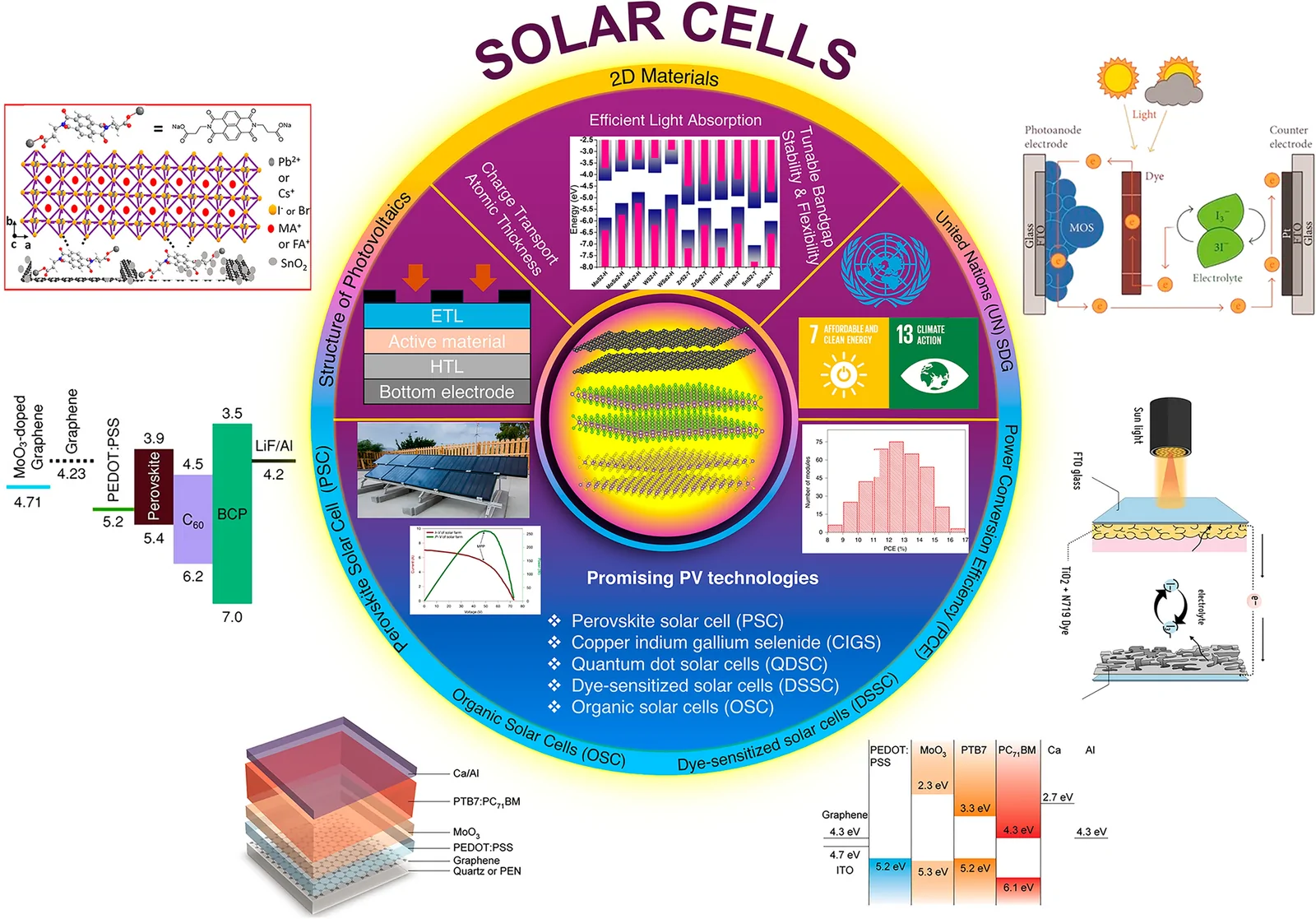
Schematic presents a detailed overview of 2D material-based solar photovoltaics and their integration into cutting-edge photovoltaic technologies. It highlights the energy-level alignments of various materials, which play a critical role in optimizing charge transport and enhancing device efficiency. These advancements demonstrate significant potential in contributing to global sustainability initiatives by enabling the development of affordable and clean energy solutions, aligned with United Nations (UN) Sustainable Development Goals (SDGs), adapted with permission from [44–51]

In this review, we begin with recent advancements in 2D materials-based photovoltaics as promising 2D materials for next-generation solar cells, focusing on their applications as ETL and HTL. The layout of 2D materials-based solar cells is explored, focusing on planar architecture, bulk heterojunction and nanocomposite configurations with their pros and cons. For PSCs, the role of 2D materials is discussed in improving perovskite crystallization, passivate defects and optimizing charge transport for improved stability and efficiency. In OSCs, 2D materials potentially reduce recombination and improve electron collection by modifying the work function and interface barrier. For DSSCs, 2D materials are explored as counter electrodes (CEs) to enhance efficiency, stability and electrocatalytic activity. Challenges include limited light absorption, defect susceptibility and scalability. In PSCs, interface engineering, energy-level alignment, perovskite composition and morphology control are addressed to discuss these limitations. For OSCs, the focus is on donor–acceptor blends with complementary properties and methods to reduce work function, such as doping, surface treatment and nanostructuring. Furthermore, long-term stability of dye and electrolyte, electrolyte leakage and development of efficient, cost-effective counter electrodes are addressed for next-generation photovoltaics. Figure 1 presents a comprehensive overview of 2D materials and their role in optimizing carrier transport and enhancing device efficiency for next-generation solar technology.

## Rise of 2D Materials

**Fig. 2:**
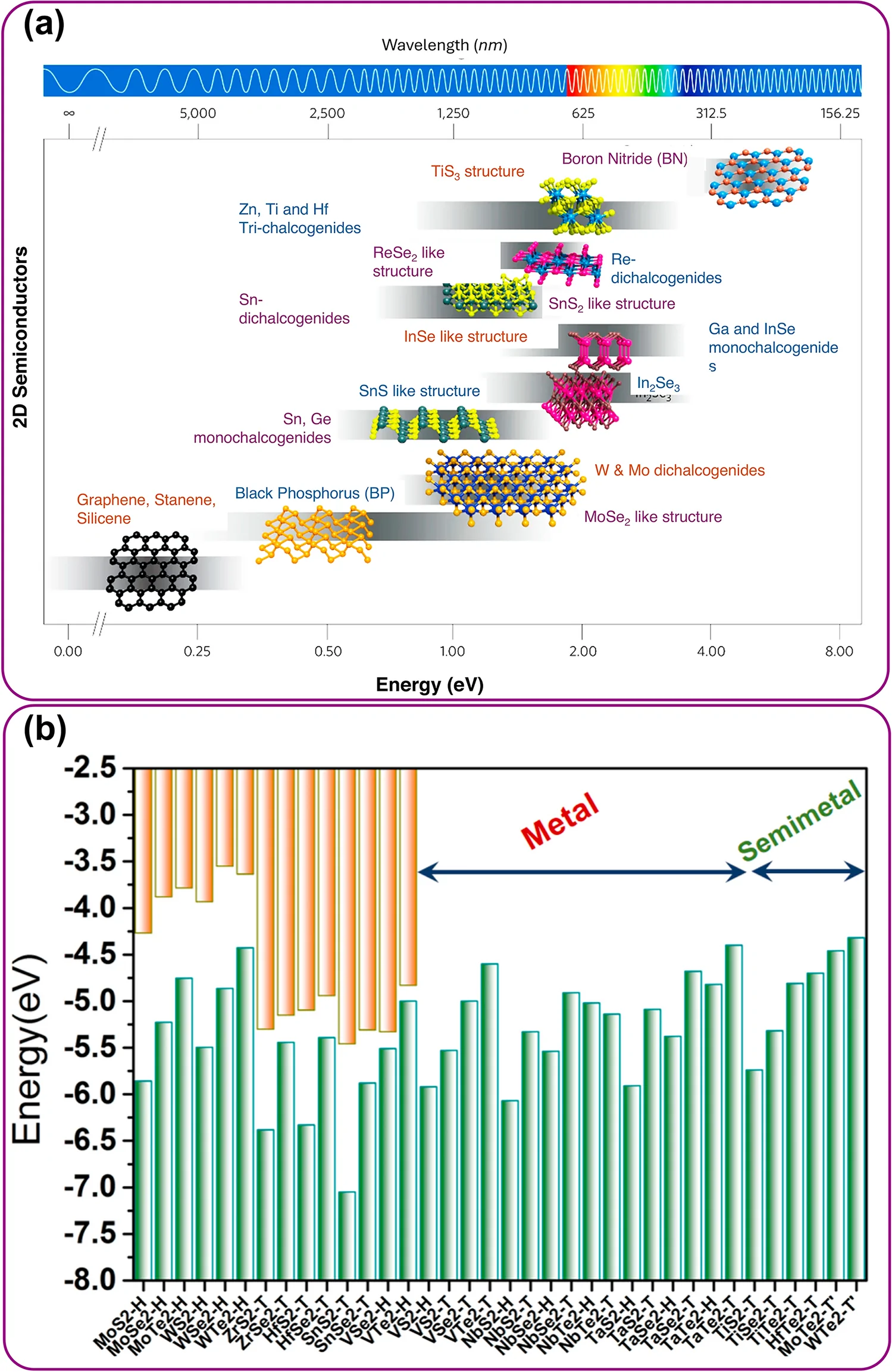
**a** Crystal structure and atomic arrangement of each material, highlighting their structural similarities and differences. The wavelength scale corresponds to specific energy levels measured in eV along the bottom and an inverse relationship between the energy and wavelength**.** The adjustable bandgap of 2D semiconductors represents the energy difference between valence and conduction band achieved by varying the number of layers, strain, or alloying, offers potential applications ranging from photovoltaics, infrared detection, solar energy harvesting and optical communication, adapted with permission of Nature Springer [53]. **b** Band structure alignment for monolayer TMDCs with valence band maximum and conduction band minimum as orange and green bars, respectively, at vacuum level 0 eV, adapted with permission of IOP Publishing Ltd. [50]

The groundbreaking isolation of single-layer graphene through mechanical exfoliation marked a pivotal moment [52]. This discovery ignited a surge of research into 2D materials, and the field continues to expand exponentially. A key driver behind this rapid growth is the immense potential of 2D materials to revolutionize various technological sectors. Their unique properties hold promises for advancements in electronics and optoelectronics, among other fields. Many 2D semiconductor materials have been mechanically exfoliated and have a remarkable range of band gaps, varying from milli-electron-volts to several electron-volts. This diversity paves the way for hundreds more to be discovered in the near future that could revolutionize the electronics field and the wide spectrum of bandgaps in 2D materials allows us to tune material properties for diverse applications. For instance, a narrow-bandgap material might be ideal for solar cells, while a wider bandgap material could be better suited for transistors. Crystal structures of various 2D semiconductor families, highlighting their similarities and differences as well as band structure alignment, are shown in Fig. 2a, b. The gray horizontal bars visually represent the range of band gaps achievable within each family. These bandgaps can be modified by varying thickness, applying strain, or creating alloys. The wide range of achievable bandgaps allows 2D semiconductors to address diverse needs across the electromagnetic (EM) spectrum [53].

Two-dimensional materials provide a unique combination of atomically smooth surfaces, tailorable work functions, and tunable carrier types that make them ideal interfacial charge transport layers in perovskite solar cells (PSCs). For efficient hole transport layers (HTLs), a high work function (~ 5.0 to 5.4 eV), p-type conductivity and strong valence-band alignment with the perovskite absorber (typically 5.4–5.9 eV) are essential [54]. A higher work function has a deeper Fermi level, which helps create a favorable energy-level offset with the perovskite valence band for hole extraction and reduces energy losses. For efficient hole extraction, precise valence band alignment at the perovskite/HTL interface is crucial. An optimal configuration typically involves a minimal energy barrier, often realized when the HTL valence band maximum (VBM) is slightly higher, i.e., more negative, closer to the vacuum level than that of the perovskite, ensuring low resistance and facilitating facile hole transfer. Alternatively, engineering a “hole cascading” energy landscape can further mitigate charge recombination. However, any substantial energy barrier at this interface would significantly hinder efficient hole extraction. Graphene derivatives doped with sulfonic or amine groups, and semiconducting TMDCs such as WSe_2_ (work function ~ 5.1 eV, hole mobility 10^1^–10^2^ cm^2^ V^−1^ s^−1^) meet these criteria while offering excellent transparency and mechanical flexibility [55].

Conversely, an effective electron transport layer (ETL) demands a wide optical bandgap (> 2.5 eV) to suppress parasitic absorption, an n-type character, and a deep conduction-band minimum (~ 4.0 eV) that matches or lies below that of the perovskite. Few-layer MoS_2_ or WS_2_, when chemically doped to enhance n-type mobility and insulating h-BN sheets (bandgap ~ 5.9 eV), fulfill these requirements, simultaneously serving as physical barriers that hinder ion migration and moisture ingress [56]. A favorable energy landscape is required to efficiently transport electrons from the perovskite layer into the ETL. The ETL conduction-band minimum (CBM) must be positioned at an energetically low level and align favorably with the perovskite's CBM, either by matching it or lying slightly below. For efficient electron transfer, the ETL CBM should be lower, i.e., more negative, closer to the vacuum level than the perovskite's CBM. This creates an “energy downhill” path, providing a strong driving force for electrons to transfer, minimizing energy barriers at the interface, facilitating facile electron collection and suppressing interfacial recombination losses. Beyond simple energy-level matching, 2D layers contribute to PSC performance by passivating interfacial defects (through Pb–S or Pb–O coordination), guiding perovskite grain growth via van der Waals epitaxy and providing ultrathin, pin-hole-free encapsulation [57].

### Graphene

**Fig. 3:**
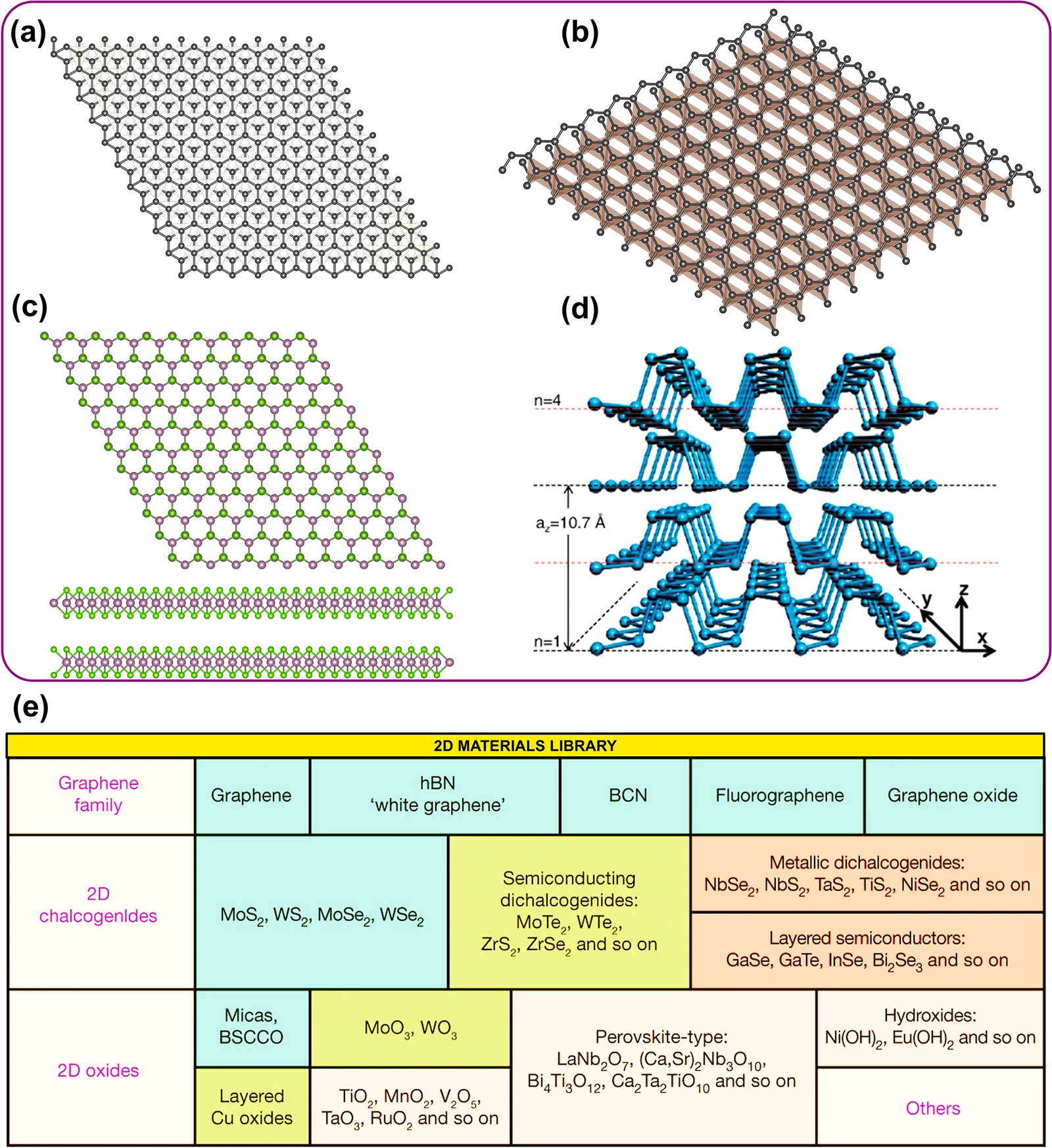
Crystal structure and library. **a**, **b** Crystal structure of graphene along different planes. **c** Structures of TMDCs and **d** lattice structure of BP and out-of-plane lattice constant a_z_ with n number of layers*,* adapted with permission of AMER PHYSICAL SOC [98].** e** The stability of monolayers under different conditions is influenced by several factors, including atomic structure and bonding, defects and impurities, interaction with the environment, thickness and temperature. Monolayers are classified based on their stability under ambient conditions (cyan for stable, green for likely stable and tan for potentially stable in an inert atmosphere), adapted with permission of Nature Springer [99]

Graphene is a remarkable combination of optoelectronic and mechanical properties, along with its strong bonding to various organic materials, making it a valuable material for high-performance organic devices. This unique synergy allows for enhanced performance in organic light-emitting diodes (OLEDs), field-effect transistors (FETs) and organic photovoltaic cells (OPVs) [46, 58]. It has an exceptional specific surface area that provides enough space for the adsorption and storage of ions and good chemical stability that ensures its long-term durability. Doping graphene with specific elements or using multilayer graphene structures could potentially improve its conductivity while maintaining some transparency and could serve as an alternative to traditional transparent conductive electrodes in solar cells. It can be combined with other light-absorbing materials to create composite structures which could improve charge transport and collection within the solar cell, while the other material handles light absorption [44, 59]. Crystal structures with different perspectives are shown in Fig. 3a, b. Graphene single-atom thick crystal shows an unusually low density of defects within the material that typically acts as scattering centers, hindering the movement of charge carriers and ultimately limiting mobility. The minimal presence of such defects in graphene allows for highly efficient charge transport. It has ambipolar field-effect behavior that allows for the continuous tuning of the carrier type by using an applied electric field. The Dirac point in graphene marks the intersection of the valence and conduction bands, resulting in a zero-band gap material. By applying a negative gate voltage, the Fermi level is shifted below the Dirac point results in a significant abundance of holes being introduced into the valence band leading to hole conduction. Conversely, a positive gate voltage elevates the Fermi level above the Dirac point, resulting in a significant abundance of electrons into the conduction band, enabling electron conduction [60].

### Transition Metal Dichalcogenides (TMDCs)

TMDCs represent one of the most extensively studied families of 2D materials after graphene, with the general formula of MX_2_, where M represents a transition metal element and X represents a chalcogen element like selenium, sulfur or oxygen, as shown in Fig. 3c [61, 62]. A key characteristic of TMDCs is their intrinsic bandgap, which falls within the visible spectrum of light and quantum confinement effects that restrict the movements of electrons due to material thinness and affect its properties. Since the material is confined in an ultrathin layer, the bandgap exhibits dependence on the number of layers and shows a transition in the bandgap nature as the thickness increases, from a direct bandgap in monolayers to an indirect bandgap in multilayers [63, 64]. These materials possess strong spin–orbit interaction due to the presence of heavy transition metals within the material atomic structure. Spin–orbit is a relativistic interaction that couples the electron spin intrinsic angular momentum to its orbital motion. Spin-orbital interaction in TMDCs causes the single valence bands to gets divided into two sub-bands with slightly different energies that result in a significant impact on the optical properties of the material [65, 66]. These families of materials had exceptionally strong exciton binding energy required to separate an exciton back into a free electron and hole. An exciton quasiparticle forms when an excited electron and a hole are bound together by electrostatic attraction and strong exciton binding energy in TMDCs makes them ideal candidates for studying the physics of excitons through photoluminescence (PL) spectroscopy even at ambient conditions. Research on these materials has unveiled fascinating light–matter interactions. One such phenomenon is the formation of charged excitons, also known as trions which consist of an e–h pair bound to an additional charge carrier and can be an extra electron or hole. Additionally, these materials exhibit valley-polarized photoluminescence in which emitted light has the same circular polarization as the absorbed light due to selective excitation of a specified valley. In this process, light with a specific circular polarization where an electric field oscillates in a circular path and the direction of rotation either clockwise or anticlockwise defines the handedness of the polarization excites electrons in a particular valley of the material band structure, leading to the emission of light with the same polarization [67–69].

### Black Phosphorous (BP) and Black Arsenic

Black phosphorus, a relatively new 2D material has gained significant interest because of its exceptional properties of high charge carrier mobility of 100 to 1000 cm^2^ v^−1^ s^−1^ that is exceeding most other 2D semiconductors [70]. It exhibits two fascinating electronic properties; (a) a tunable bandgap and (b) pronounced in-plane anisotropy. The bandgap of BP covers a wide range of the EM spectrum, extending from the visible to the mid-infrared region, enabling it to interact with diverse light wavelengths. Unlike most 2D materials that show strong anisotropy between in-plane and out-of-plane directions, BP exhibits an additional unique property, i.e., in-plane anisotropy in which its optical and electrical properties vary depending on the direction within the plane of the material [71–73]. The exceptional in-plane anisotropy of BP is due to its unique atomic bonding compared to other layered materials like graphite. In graphite, each carbon atom forms three planar bonds with its neighbors using *sp*^2^ hybridization, while in the case of BP, four valence orbitals combine to form four *sp*^3^ hybrid orbitals suitable for forming strong tetrahedral bonds where each phosphorus atom bonds to three neighboring atoms in a tetrahedral arrangement as shown in Fig. 3d [74, 75]. This distinct bonding scheme leads to a puckered honeycomb lattice structure, unlike the planar structure of graphite. The puckered honeycomb lattice structure in BP is the foundation for its highly anisotropic band structure. An anisotropic band structure refers to variations in the energy levels of electrons depending on their direction of movement within the material. This unique structure leads to BP exhibiting pronounced in-plane anisotropy in its optical, thermal, electrical and mechanical properties. This characteristic stands in stark contrast to other layered materials like graphene, boron nitride and molybdenum or tungsten-based TMDCs which display minimal in-plane anisotropy [76, 77]. It exhibits a remarkable optical property of linear dichroism where light absorption depends on the relative orientation between the material crystal lattice and incident linearly polarized light. It absorbs light differently based on the direction of the light electric field relative to its atomic arrangement. This linear dichroism has significant consequences for several aspects of BP like Raman spectra which used to study the vibrational modes in materials and can be influenced by the direction of light polarization due to dichroism and plasmonic and screening effects that refers to collective oscillation of electrons on the material surface and how effectively the material screens electric fields can be affected by the polarization of light and photoresponse; the way BP respond to light, including the generation of electrical current or light emission can be dependent on the polarization of the incident light. Black arsenic (b-As), a less-explored allotrope of arsenic, exhibits remarkable anisotropies in its thermal, electronic and electric transport properties along its in-plane principal axes-armchair (AC) and zigzag (ZZ) directions. These anisotropies surpass or match those of any other known 2D materials, such as TMDCs and graphene, making b-As an exciting candidate for future research and potential device applications. Due to distinct atomic arrangements and bonding properties, the electronic band structure of b-As along the AC and ZZ directions is different and charge carriers will experience different mobilities and resistance based on current flow direction. These anisotropic properties are potentially modulated by the current flow directions and could be used in novel thermoelectric and electronics applications. b-As has a similar puckered layered structure as BP and exists in three crystalline allotropes, i.e., black arsenic (b-As), yellow arsenic (y-As) and gray arsenic (g-As). g-As is the most stable allotrope of arsenic and has metallic properties as compared to y-AS that exhibits insulating properties and had waxy appearance [78]. Both the b-AS and BP have structural similarities and share attributes like layer-dependent behavior and tunable electronic properties that facilitate the integration into existing technologies. The in-plane anisotropy is expected to inspire new breakthroughs and design for more sustainable and efficient energy devices. Table 1 summarizes the bandgap and charge carrier mobility of various 2D materials.

**Table 1:** Bandgap and carrier mobility of various notable 2D materials

Material	Mobility (cm^2^ v^−1^ s^−1^)	Bandgap (eV)	Refereces
WSe_2_	140–500	1.2–1.7	[79–81]
TiS_2_	7.24	0.02–2.5	[82, 83]
SnS_2_	50–230	2.18–2.44	[84, 85]
MoS_2_	10–200	1.2–1.8	[79, 86]
Graphene	~ 2 × 10^5^	~ 0	[87, 88]
Ti_2_CO_2_	~ 400	0.91	[89]
WS_2_	43–234	1.3–2.1	[90]
HfCO_2_	~ 1100	1.79	[91]
Zr_2_CO_2_	~ 600	1.76	[89]

### World of Elemental 2D Materials Extends beyond TMDCs, BP and Graphene

Group XIV of the periodic table following carbon holds elements capable of forming similar single-atom-thick materials. Since graphene structures are built from carbon atoms, silicon (silicene), germanium (germanene) and tin (stanene) can form their own 2D analogs [92]. However, unlike graphene flat honeycomb lattice, these elements exhibit a distinctive buckled atomic arrangement. Compared to carbon, silicon, germanium and tin possess stronger spin–orbit coupling. This enhanced interaction between an electron spin and its orbital motion significantly impacts the electronic band structure of their corresponding 2D materials (silicene, germanene and stanene) compared to graphene. Consequently, a bandgap is predicted to open in these materials and estimated values for these bandgaps are around 300 meV for stanene, 20 meV for germanene and 2 meV for silicene [93, 94]. While successfully fabricated, these narrow-bandgap 2D semiconductors (silicene, germanene and stanene) present significant hurdles for characterization due to their growth methods. A collection of 2D materials with their stability at room temperature are shown in Fig. 3e. Epitaxial growth on metallic surfaces within ultrahigh vacuum environments limits their exposure to standard analysis techniques [95, 96]. Additionally, these metallic substrates significantly influence the intrinsic electronic properties of 2D materials and limits the exploration of their true potential and applications in areas like mid and near-infrared optoelectronics. However, a recent breakthrough has emerged where the encapsulation of silicene involves the removal of material from the high vacuum environment and forms functional devices which paves the way for understanding of these 2D materials and their potential applications for cutting-edge optical devices [97]. By overcoming the limitations of environmental instability arising from the initial growth methods, the encapsulation technique unlocks a new era for studying this class of 2D materials which opens the door for experimental research where a significant gap in experimental data currently exists.

### MXenes as an Emerging Material for Photovoltaic Applications

MXenes, a rapidly emerging class of 2D transition metal carbides, nitrides and carbonitrides, have attracted significant attention due to their exceptional physicochemical properties. First discovered in 2011, MXenes have the general formula *M*_*n*+1_*X*_*n*_*T*_*x*_, where M is a transition metal, X is carbon and/or nitrogen, and *T*_*x*_ represents surface terminations such as –OH, –F, or –O. These materials offer high electrical conductivity, tunable work functions, good hydrophilicity and solution processability, making them ideal for integration into flexible and printable optoelectronic devices [100, 101]. Their layered structure, which facilitates interfacial contact and ion transport, combined with their strong light absorption and capability for work function modulation, makes MXenes promising candidates for improving the performance of photovoltaic technologies, especially as charge transport materials, interfacial modifiers, or transparent conductive electrodes [102].

In PSCs, MXenes have demonstrated promising functionality as both ETLs and HTLs, as well as interfacial modifiers to enhance charge extraction and suppress recombination. Their tunable work function (typically in the range of 4.2–5.6 eV, depending on surface terminations) enables effective energy-level alignment with perovskite and transport layers, improving open-circuit voltage and fill factor. Furthermore, their excellent conductivity and passivation ability can enhance charge mobility while reducing trap states and hysteresis [103, 104]. For example, Ti_3_C_2_T_x_ MXene has been shown to increase device stability by serving as a buffer layer that blocks ion migration and prevents moisture ingress. Additionally, MXenes can improve the crystallinity of the perovskite layer when incorporated into the precursor solution or used as a substrate, resulting in higher power conversion efficiency and improved operational stability [105, 106].

MXenes have also been explored in OSCs, where their high conductivity and tunable surface chemistry allow them to function effectively as electrode modifiers, interfacial layers, or even active additives in the photoactive layer. In inverted OSC architectures, MXenes such as Ti_3_C_2_T_x_ can be used to modify the work function of electrodes like ITO, facilitating efficient electron extraction and reducing energy barriers at the interface [107, 108]. Moreover, the incorporation of MXenes into the donor–acceptor blend has shown to improve phase separation and charge carrier transport, leading to reduced recombination losses. Their mechanical flexibility and compatibility with low-temperature, solution-based processes also make them particularly attractive for flexible and printed OSCs. Recent studies have reported that MXene-based interlayers can significantly improve both the short-circuit current density and fill factor, thereby enhancing the overall performance and stability of OSC devices [109].

## 2D Materials Integration in Solar Cell

The integration of 2D materials in solar cells marks the significant advancement in PV technology, offering unique properties that improve the performance and efficiency of devices. With their atomically thin structure, 2D materials such as TMDCs, graphene and other layered materials provide improved electrical conductivity and light absorption capabilities. These materials can be utilized in various configurations, including charge transport layers, active layers and interface modifiers, enabling the development of novel solar cell architectures [110–112]. This section explores various solar cell configurations using 2D materials, highlighting their potential to enhance energy conversion efficiency. Despite various layouts, these cells share common components that influence performance. The details of these components and their effects are outlined below as.

### Top Electrode

The top electrode in 2D material-based solar cells serve two important functions. **a) Light Transmission**, which allows a significant portion of the incoming sunlight to pass through and reach the light-absorbing layer within the device that ensures efficient capture of solar energy for conversion into electricity. **b) Current Collector;** once light is absorbed and generates electron–hole pairs, it acts as a collector for the photogenerated current. Indium tin oxide (ITO) is commonly used as a top electrode because of its high transparency across a broad range of wavelengths, allowing a significant amount of sunlight to penetrate the solar cell and excellent conductivity enables the efficient collection of the generated holes and facilitates their flow into the external circuit. However, currently there has been ongoing research to actively explore alternative materials for the top electrode to potentially overcome some limitations of ITO, such as its scarcity and relatively high cost which might offer even better transparency, conductivity, or compatibility with specific device architectures [113–115].

### Hole Transport Layer (HTL)

The HTL is crucial for the efficient operation of 2D material-based solar cells. The HTL material is chosen for its ability to selectively transport holes generated within the active layer to ensure minimal transport of electrons in the opposite direction and efficient separation of these charges for maximizing solar cell performance. The HTL layer also facilitates the movement of holes toward the top electrode and offers a low energy barrier for hole transfer, allowing for smooth and efficient extraction of these charges from the active layer. The selection of an appropriate HTL material depends on multiple key factors that include **(a) Energy-Level Alignment:** The bandgap and work function of the material is chosen to ensure proper alignment with the energy levels of both the top electrode and active layer which allows for efficient transfer of holes from the active layer to the HTL and then to the top electrode for current collection. **(b) High Hole Mobility:** The material should have high hole mobility to minimize resistance to hole transport which ensures efficient movement of holes through the HTL with minimal energy loss. **(c) Stability and Compatibility:** The material must be chemically stable and compatible with the other solar cells components to ensure long-term device performance and avoid environmental degradation [116–118].

### Active Layer

The active layer is the core component of a 2D material-based solar cell, responsible for converting light energy into electricity which is designed to efficiently absorb incoming sunlight that excites electrons within the material to higher energy states. In solar cells using 2D materials, the active layer is typically comprised of one or more materials like graphene, BP or TMDCs that have unique properties for light absorption. The selection or combination of different 2D materials can tailor the active layer to absorb a broader range of solar spectrum, maximizing the light capture efficiency. Certain 2D materials exhibit exceptional light–matter interaction properties, allowing them to efficiently capture incoming photons and generate excitons.

### Electron Transport Layer (ETL)

The ETL in 2D materials-based cells facilitates the movement of electrons toward the bottom electrode of the solar cell and offers a low energy barrier for electron transfer, allowing for efficient and smooth extraction of these charges from the active layer. The ETL material is chosen for its ability to selectively transport electrons generated within the active layer that minimize the movement of holes in the opposite direction. Effective separation of these charges is crucial for maximizing solar cell performance. The appropriate ETL material is selected based on several key factors. **(a) Energy-Level Alignment:** The work function and bandgap of the ETL need to be carefully chosen to ensure optimal alignment with the energy levels of the bottom electrode and the active layer which allows for the efficient electron transfer to ETL and then to the bottom electrode for current collection. **(b) High Electron Mobility:** The ETL material possess a high electron mobility to minimize any resistance to electron transport which ensures efficient movement of electrons through the ETL with minimal energy loss. **(c) Chemical Stability and Compatibility:** The ETL material must be compatible and stable with the other components of the solar cell to ensure excellent performance and prevent degradation [119–121].

### Bottom Electrode

The bottom electrode serves as the final electron collector and completes the electrical circuit. Once electrons are transported through the ETL, the bottom electrode acts as a sink to collect them and creates electrons flow from the active layers to the circuit. The ohmic contact with the ETL allows for efficient transfer of collected electrons, ensuring minimal energy loss during electron transfer. The selection of an appropriate material for the bottom electrode is crucial for optimal solar cell performance and depends on many key factors. **(a) High Electrical Conductivity:** The material should possess excellent electrical conductivity to minimize resistance to electron flow which ensures efficient collection and electrons transport from ETL to the external circuit. **(b) Work Function:** The work function of the bottom electrode needs to be carefully chosen to ensure an ohmic contact with the ETL for efficient electrons transfer from the ETL to the electrode, minimizing energy barriers. **(c) Chemical Stability:** The bottom electrode material must be stable with the other components to prevent degradation over time and ensure long-term device performance. Common materials used for bottom electrodes in 2D materials-based cells include aluminum (Al) and calcium (Ca). These materials offer a good balance of conductivity, work function and stability. However, ongoing research seeks alternative materials that could provide superior properties and enhanced compatibility with specific device architectures [115, 122–124].

**Fig. 4:**
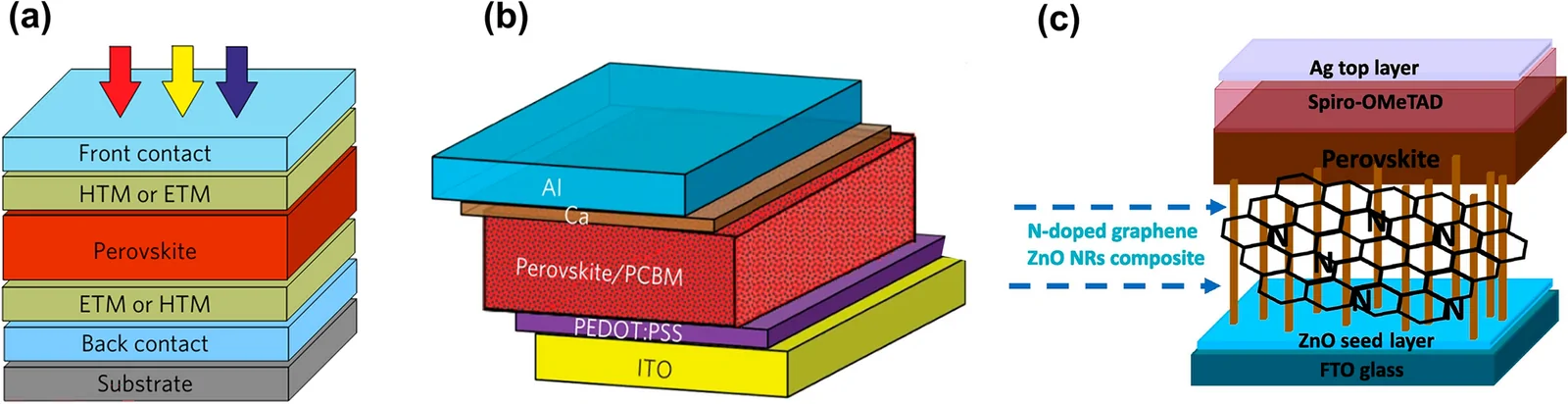
2D materials-based solar cells layout. **a** Planar architecture; a simple layered design and easy to fabricate in which each layer is stacked on top of each other, adapted with permission of Nature Springer [132]. **b** Bulk heterojunction architecture; it uses 2D materials blend donor–acceptor materials to facilitate efficient exciton dissociation; thereby, intermixed materials create a large interfacial area for exciton separation to improve charge separation and generation of free carriers, adapted with permission of Nature Springer [133]. **c** Nanocomposite architecture; it uses 2D material nanoparticles embedded in a matrix for enhancing charge transport and light absorption. Material selection and morphology control are crucial for efficient exciton dissociation and minimizing recombination to achieve high efficiencies, adapted with permission of ELSEVIER [134]

The **planar architecture** is the most basic layout for solar cells that adopts a layered approach where all the functional components are vertically layered on top of one another in a well-defined sequence as shown in Fig. 4a. This structure features a relatively simple design, making it easier to fabricate compared to more complex alternatives and allowing for potential scalability in the manufacturing process [125, 126]. However, this simpler design does have certain limitations that include **(a) Limited Light Path Length:** In this design, light traverses the entire device stack before reaching the active layer which limits the overall light absorption efficiency, especially for thicker layer and **(b) Charge Recombination Losses:** The travel distance for charges within the device can be relatively longer in planar architecture which increases the possibility of these charges recombining before reaching their respective electrodes, leading to energy losses and reduced efficiency.

The **bulk heterojunction** architecture offers a promising approach for 2D materials-based solar cells by incorporating a unique active layer that is a blend of intermixed donor-accepter materials. Donor material efficiently absorbs photons and generates excitons while the acceptor material has a higher electron affinity than the donor which facilitates the separation of excitons into free holes and electrons by accepting the excited electron from the donor. The acceptor and donor material within the heterojunction is intermixed at the nanoscale to create a large interfacial area between two materials for efficient exciton dissociation as shown in Fig. 4b. Excitons have a finite diffusion length within the active layer before they recombine, and intermixing ensures a high probability of excitons reaching a donor–acceptor interface before recombination. At the interface, the energy difference between the acceptor and donor materials allow for efficient transfer of the excited electron from the donor to the acceptor and separation creates free electrons and holes that are collected by their respective electrodes. The large interfacial area and short diffusion lengths in the bulk heterojunction design promote efficient exciton dissociation, leading to improved charge separation and generation of free carriers. However, there are some challenges associated with this architecture. The performance of these types of cells is highly dependent on the morphology of the donor and acceptor materials within the blend, and optimizing this morphology is required to ensure a good balance between interfacial area and charge transport pathways. Therefore, careful selection of donor and acceptor materials is essential for excellent electronic compatibility and efficient charge transfer at the interface [127, 128].

The **nanocomposite architecture** presents an innovative approach for designing the active layer in 2D material-based solar cells. It incorporates a composite material formed by nanoparticles and matrix material. Nanoparticles are comprised of 2D materials like molybdenum disulfide (MoS_2_) or other light-absorbing materials like fullerenes while matrix material surrounds and embeds the nanoparticles and often consists of a conductive polymer or inorganic scaffold. These incorporated nanoparticles can improve overall light capture efficiency within the active layer and provide efficient pathways for transporting either electrons or holes depending on the specific design that promotes efficient separation and collection of photogenerated charges as shown in Fig. 4c. This design approach offers flexibility in material selection for both nanoparticles and the matrix, allowing for customization of charge transport and light absorption properties. By optimizing the combination of materials and their arrangement within the nanocomposite, we can achieve enhanced light capture and efficient charge transport, leading to potentially higher solar cell efficiencies. However, there are some challenges associated with this architecture like controlling morphology and optimizing the size, distribution and interface between nanoparticles and the matrix is important for efficient transport of charges and exciton dissociation. Depending on the materials and their arrangement, there exists a risk of increased charge recombination at the interfaces within the nanocomposite and careful design and material selection could potentially minimize this recombination for improved device performance and efficiency [129–131].

## Role of 2D Materials in Photovoltaics

2D materials have been at the center stage of research for more than a decade due to their incredible optics and electronic properties. Traditionally, solar cells have relied on silicon wafers, while effective at capturing sunlight, their thickness and weight make them bulky and unsuitable for certain applications like incorporating them into wearable electronics. Additionally, silicon wafers are rigid and inflexible, limiting their use on curved surfaces or for applications that require flexibility. However, the trend in photovoltaics is moving toward thinner and lighter materials. Since 2D materials are just a single or few atomic layers thick, they are naturally lightweight and flexible. These properties open the door for new solar cell applications, such as incorporating them into fabrics for wearable electronics or even curving them around surfaces.

### Role of 2D Materials in Perovskite Solar Cells (PSCs)

#### Optimizing Defect Passivation and Crystallization Control in PSCs via 2D Materials

**Fig. 5:**
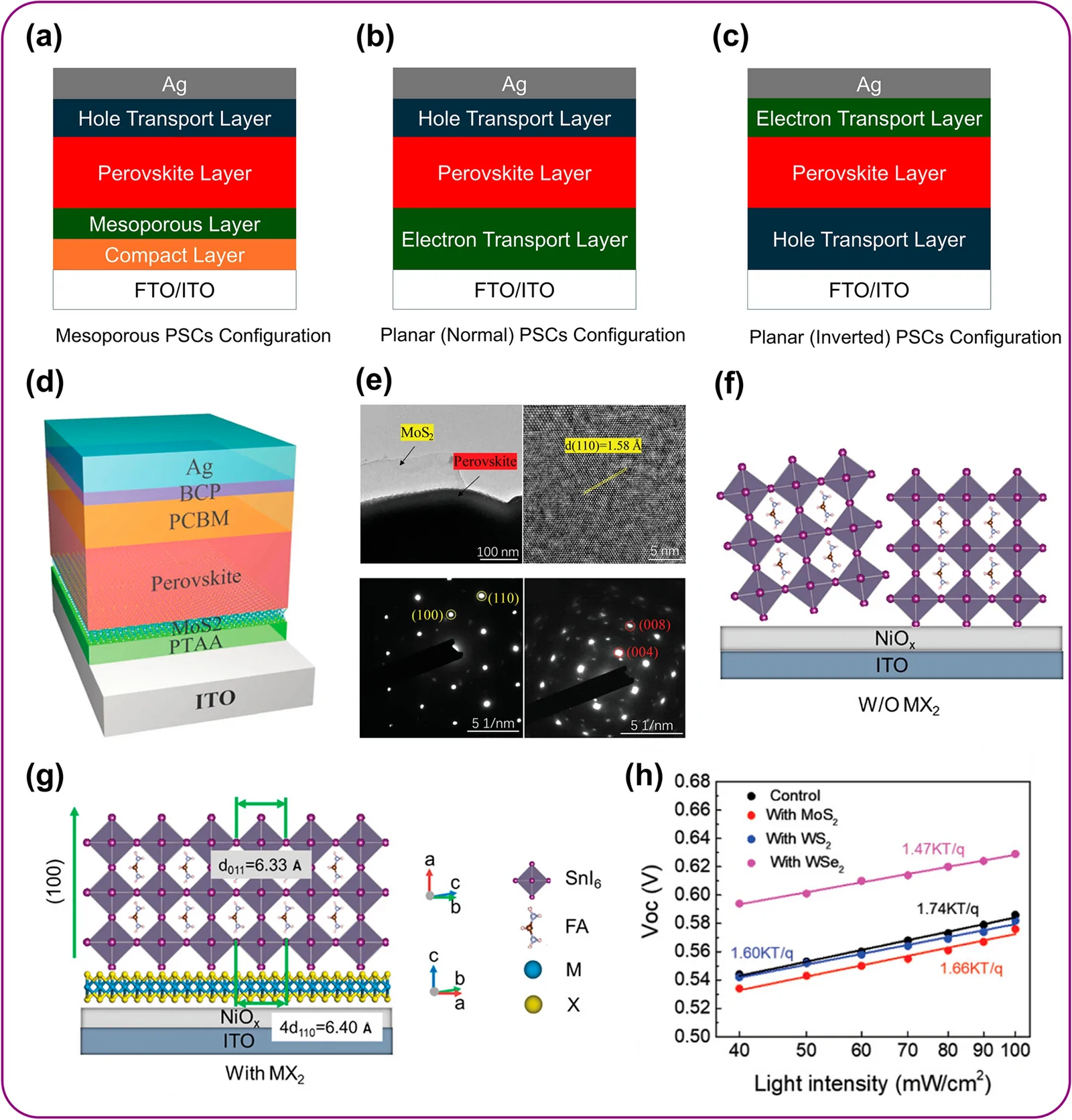
Optimization of crystallization and passivation of defects. **a** Mesoporous PSC; a mesoporous scaffold material between the bottom electrode and perovskite layer enhances light absorption and charge transport. **b** Planar (Normal) PSC; this structure directly contacts the perovskite layer with both electrodes (top and bottom) that allows the efficient charge collection. **c** Planar (Inverted) PSC; this structure reverses the electrode positions in a planar (normal) design that is advantageous for air-sensitive organic HTLs by protecting them from degradation and potentially improving charge collection at the perovskite-HTL interface. **d** Structure of an inverted planar perovskite solar cell with MAPbI_3_ deposited on MoS_2_ flakes where PTAA and PCBM serve as HTL and ETL, respectively.** e** TEM and HRTEM images of MoS_2_ with a MAPbI_3_ perovskite layer, along with SAED patterns of MoS_2_ and perovskite, respectively, **d**, **e** adapted with permission of Wiley [138]. **f**, **g** FASnI_3_ grain growth on NiO_x_ w/o MX_2_ and vdW epitaxial growth of FASnI_3_ on a MX_2_ (WSe_2_) surface and WSe_2_ energy-level alignment with FASnI_3_ facilitate efficient charge carrier transfer and cascaded band structure with WSe_2_ conduction band lower than FASnI_3_ promoting hole transfer from perovskite to WSe_2_. **h** V_OC_ dependence on light intensity shows variations in recombination mechanisms with WSe_2_ lowest slope of 1.47 indicating the strongest suppression of trap-assisted recombination, likely due to its favorable band alignment, **f–h** adapted with permission of Wiley [139]

Metal halide perovskites (MHPs) have emerged as a highly promising class of light-harvesting materials for photovoltaics due to their broad-spectrum absorption, tunable bandgap and long carrier diffusion lengths, which allow photogenerated charge carriers to travel longer distances before recombining, ultimately supporting efficient current generation [135, 136]. The charge transport interlayers act as critical components influencing both the device photovoltaic performance and its durability over time. It efficiently extracts photogenerated charges from the perovskite layer and minimizes unwanted recombination losses within device. It facilitates efficient charge transfer by aligning bandgaps with respective valence and conduction bands of perovskite absorber. PSC can be fabricated in various architectures, each influence device performance, durability and applicability. These are mainly classified into III types: **(a) Mesoporous PSC:** This structure utilizes a mesoporous scaffold material that is sandwiched between the bottom electrode and the perovskite layer. It helps in efficient light absorption and facilitates charge transport within the perovskite layer due to large surface area of mesoporous layer. **(b) Planar (Normal) PSC:** In this device structure, perovskite layer directly contacts the top and bottom electrodes that offers more direct pathway for charge collection compared to mesoporous structure. **(c) Planar (Inverted) PSC:** In this device design, perovskite layer inserted between electrodes same as planar (normal). However, electrodes position is reversed with the ETL layer deposited on top of the perovskite and HTL layer on the bottom. This structure offers advantages for air-stable material like organic HTL, which are more susceptible to degradation when deposited directly on perovskite and may also improve charge collection at the perovskite–HTL interface. These device structures of different configurations are shown in Fig. 5a–c.

Despite the advantageous properties of MHP, several fundamental and practical challenges must be addressed before MHP-based PSCs can achieve widespread commercial adoption. These challenges include their inherent instability under environmental stressors such as heat, humidity and UV radiation, as well as the prevalence of deep-level defects at grain boundaries and interfaces that significantly limit device efficiency and long-term durability.

To overcome these limitations, 2D materials such as TMDCs (MoS_2_, WS_2_, WSe_2_) and graphene derivatives have been explored extensively due to their unique properties of high carrier mobility, excellent surface smoothness, tunable electronic structures and robust chemical stability [137]. When incorporated as interfacial modifiers or charge transport layers, these 2D materials not only aid in band alignment and charge transport but also serve as multifunctional layers for defect passivation, which plays a critical role in enhancing photovoltaic performance and operational stability. One key mechanism is chemical passivation via ion bonding, where specific atomic species in the 2D material form strong coordination bonds with undercoordinated ions at the perovskite surface. For instance, sulfur atoms in MoS_2_ QDs can chemically bind to uncoordinated Pb^2+^ ions, forming Pb–S bonds that passivate electronic trap states and suppress non-radiative recombination losses. Similarly, functional groups such as hydroxyl, carboxyl, or amine on functionalized reduced graphene oxide (f-RGO) or graphene oxide (GO) can chemically interact with both Pb^2+^ and halide vacancies, mitigating surface defects. These chemical interactions are crucial for increasing the open-circuit voltage (V_oc_) and fill factor (FF) of the device by minimizing charge recombination at the interface [138].

Another mechanism is grain orientation control through van der Waals epitaxy. TMDCs such as MoS_2_ and WSe_2_, with their atomically flat surfaces and low lattice mismatch with perovskite crystals, can act as templates that guide the nucleation and epitaxial growth of perovskite films. In the work of Tang et al., MAPbI_3_ films were grown on MoS_2_ flakes via solution-phase deposition, resulting in strong in-plane lattice coupling between the two materials [138]. This interaction facilitated the formation of uniform, large-grained perovskite films with fewer grain boundaries and reduced defect densities. Device structure, high-resolution TEM (HRTEM) and selected area electron diffraction (SAED) confirmed the crystalline alignment between MoS_2_ and the perovskite layer as shown in Fig. 5d, e, directly correlating with improved charge transport and reduced trap-assisted recombination. Similarly, in another study, WSe_2_ flakes were employed as growth templates for FASnI_3_ perovskites [139]. The van der Waals interaction between WSe_2_ and the perovskite layer promoted grain alignment along the (100) plane, enhancing film crystallinity and facilitating efficient charge transfer across the interface as shown in Fig. 5f, g.

The third mechanism involves physical barrier layer formation. Certain 2D materials, such as f-RGO, graphene and h-BN, can form compact and continuous interfacial layers that act as impermeable barriers against moisture, oxygen and migrating ions. These barrier layers not only protect the perovskite from environmental degradation but also suppress detrimental ion migration, such as halide movement or metal diffusion, which is known to cause hysteresis and instability in PSCs. By preventing these degradation pathways, such barrier-forming 2D materials contribute significantly to improving both device longevity and operational reliability [24, 140].

The impact of these mechanisms is quantitatively demonstrated through *J*–*V* and *V*_oc_-light intensity measurements. For instance, devices incorporating TMDCs interlayers (MoS_2_, WS_2_, WSe_2_) showed clear reductions in the slope of the *V*_oc_ versus light intensity plot from 1.74 (kT q^−1^) for the control device to 1.47 (kT q^−1^) for the WSe_2_-based device, indicating the suppression of trap-assisted recombination as depicted in Fig. 5h. These enhancements in interfacial quality and defect control directly contribute to improved PCE, *V*_oc_ and FF.

Collectively, these findings confirm that 2D materials contribute to perovskite performance enhancement not only through charge transport and band alignment but also through multiple defect passivation strategies, including chemical interaction, crystallographic templating and physical encapsulation. Table 2 provides a detailed comparison of various functionalities observed in PSCs that incorporate 2D materials.

**Table 2:** Summary of efficiency, durability and functional roles of 2D Materials in PSCs

Year	Device Structure	Durability	Functionality	PCE (%)	References
2022	ITO/SnO_2_/perovskite/QD/GO/spiro-OMeTAD/Au	Retaining 90% of its efficiency for 1000 h at 60 °C in ambient air	Function as multifunctional interface modulator, anchor CsPbBr_3_ to enhance charge transport, optimize energy band alignment, passivate surface defect and act as barrier against ion and moisture diffusion	18.55	[141]
2022	ITO/PTAA/CsPbI_3_/Ti_3_C_2_T_x_/CPTA/BCP	Preserving 85% of PCE for 1000 h with 85% relative humidity	Acts as a moisture barrier, enhances charge separation via improved interfacial electric field and optimizes charge extraction at ETL/perovskite interface	19.69	[142]
2022	PTAA/BABr + MAPbl_3_/PCBM/ZnO	Retained 62% of its initial PCE after 1000 h and maintained 64% of its initial performance thermal stability after 500 h	Function as surface passivation layer with controlled orientation, reduce trap density, enhance charge transport and improve stability through anisotropic crystal engineering	21.40	[143]
2022	Mp-TiO_2_/CsFAMA/F-BP/spiro-OMeTAD	Maintained 95% of its efficiency at room temperature after 30 days	Enhance antioxidant properties, strong P-Pb coordination and interaction reduce trap states	22.06	[144]
2022	PEA2GAPb_2_I_7_ + FA_0.6_MA_0.4_Pb_0.6_Sn_0.4_I_3_/C_60_/BCP	Maintained 82% of its respective maximum PCE after 1830 h under continuous operation in N_2_	Formed mixed bulky cation additives, act as a defect passivation layer in Sn–Pb narrow-bandgap, enhances structural quality, reduce dark carrier density, increase carrier lifetime and improve stability	22.10	[145]
2022	TiO_2_/GAMA_5_Pb_5_I_16_/spiro-OMeTAD	Maintained 94% of its initial efficiency after 1200 h under ambient condition and relative humidity of 25%	Serve as the active layer with enhanced stability, improve phase purity and crystallinity, reducing trap density and boosting charge mobility	22.26	[146]
2022	SnO_2_-Mxene + Ti_3_C_2_T_x_ /(PEA)_2_MA_3_Pb_4_I_13_/spiro-OMeTAD	Maintained 90% of its initial value, at 30% relative humidity for 500 h	Serve as ETL to enhance interfacial contact and passivate defects at SnO_2_/perovskite interface, regulate SnO_2_ dispersion and promote vertical growth of perovskite with reduced interfacial stress	23.07	[147]
2022	FTO/SnO_2_/CsFAMA/Cs_3_TbCl_6_ QDs/spiro-OMeTAD:BPQDs	Preserved 88% of its PCE after 2520 h with 30% relative humidity	Regulate energy levels, passivate ionic defects and fill grain boundaries Enhance hole mobility and conductivity within HTL and overall interface optimization and stability	23.49	[148]
2022	SnO_2_/perovskite/Cs_3_TbCl_6_ QDs/BPQDs	Retained 88% of original PCE after storage in ambient air with relative humidity of 30% within 2520 h	Modify HTL for improved charge extraction and interfacial contact Fill grain boundaries, regulate energy levels, reduce defect density and passivate ionic defects	23.49	[148]
2022	NiO_x_/FPEA + Cs_0.05_FA_0.85_MA_0.1_PbI_3_/PCBM/BCP	Unencapsulated device retained 100% of its initial PCE around 50% relative humidity for over 1000 h	Bulkier organic ligands slow 2D formation and promote growth of wider RDPs Mitigate electron blocking at interfaces and improved humidity stability	23.91	[149]
2022	FTO/SnO_2_/Nb_2_CT_x_/CsFAMA/Nb_2_CT_x_/spiro-OMeTAD/Ag	Retaining 93% of its efficiency after 1500 h	Dual interfacial modifiers at perovskite/CTL interface enhance carrier mobility, reduce energy-level mismatch and facilitate hole transport via oxygen terminal groups	24.11	[150]
2022	2PACz/OALI + Cs_0.03_(FA_0.9_MA_0.1_)_0.97_PbI_3_ /C_60_/BCP	Passed industrial damp-heat test and retained 95% of its PCE after 100 h at 85 °C temperature and 85% relative humidity	Used at the electron-selective interface, tailoring the number of octahedral inorganic sheets enables effective surface passivation, reduce trap states and suppress ion migration	24.30	[151]
2022	SnO_2_/NPMA + mixed perovskite/spiro-OMeTAD/MoO_3_	Unencapsulated device maintains 98% of its initial PCE after 1500 h by maximum power point tracking under continuous light irradiation	Enhance film quality by enlarging grain size, reducing grain boundaries defects and suppressing ionic diffusion	24.37	[152]
2022	SnO_2_/(BA)_4_AgBiBr_6_ + mixed perovskite /spiro-OMeTAD/MoO_3_	Maintained 90% of initial PCE under continuous heating after 1000 h	Serve as type-I heterojunction barrier, suppress trap-assisted recombination at the interface and iodide ion diffusion from perovskite to metal electrode	24.48	[153]
2023	FTO/TiO_2_/CsPbBr_3_/WS_2_/AgI_5_S_8_/Carbon	Maintained over 93% PCE for 720 h with high humidity and temperature	Serve as HTL in CsPbBr_3_ and offers lattice matching and type-II band alignment and defect passivation	10.24	[154]
2023	TiO_2_/CsPblBr_2_/Ti_3_C_2_Tx-Patched-GO	Maintained nearly the same performance at 25 °C and 10% relative humidity for over 2376 h	Serve as a multifunctional perovskite film plaster to regulate interfacial energetics and passivate defects in carbon-based PSCs Enhance energy-level alignment, charge transport and lattice stability through chemical bonding	15.04	[155]
2023	PTAA/PEDOT: PSS/FPEA/PEA/PC_61_BM	Maintained good thermal stability and retained 90% of their initial efficiency after 720 h	Act as novel spacer cation in quasi-2D RP to enhance dipole-octahedra interaction, improve crystallinity, stabilize mixed and α-FAPbI_3_ phases, optimize energy-level alignment, long carrier diffusion length and reduced trap density	16.77	[156]
2023	PEDOT:PSS/(SeMA)_2_MAPb_2_I_7_ /PDTL/PCBM:BCP	Retained its original efficiency in ambient condition and 5% relative humidity for 1008 h	Function as a selenophene-based spacer to enhance film quality and orientation Passivate surface defects, densify ETL and promote efficient electron extraction	19.03	[157]
2023	NiO_x_/g-C_3_N_4_/L-C_3_N_4_/PEAI/PCBM/BCP	Maintained 80% of its original PCE for 300 h of continuous operation	Function as interfacial layer between NiO_x_ HTL and perovskite Suppress charge carrier recombination and defective charge accumulation by improving photoinduced charge transfer	19.33	[158]
2023	SAM/PVK/PCBM/BCP	Retained excellent mechanical durability, preserving 93% of original efficiency after 1000 bending cycle at a 5 mm radius Retained 82% of initial PCE after 1000 h of aging	Serve as a seed layer within 3D perovskite to enhance built-in electric field, improve exciton dissociation and crystallization of films, reduce hole transport barrier, facilitates highly oriented homogeneous crystal growth	23.00	[159]
2023	ITO/MeO-2PACz/CsFAMA:Gr /C_60_/BCP	Retaining 938% of PCE after 1000 h	Anchor excess Pbl_2_ to control its adverse effects, passivate grain boundaries, reduce charge recombination and enhance electron extraction, improve long-term thermal and operational stability	23.70	[160]
2023	SnO_2_/Ti_3_C_2_Cl_x_/perovskite/0-TB-GDY/spiro-OMeTAD	Unencapsulated cells retained 92% of their initial PCE after 1464 h under ambient air and 80% retention observed after 1002 h of thermal exposure at 85 °C	Improved charge carrier extraction, enhanced energy band alignment due to significantly inhibited non-radiative recombination and passivated the perovskite/ETL and perovskite/HTL interfaces	24.86	[161]
2023	TiO_2_/Pbl_4_/amidino-based Dion-Jacobson/spiro-OMeTAD	Retained 97% of its efficiency without encapsulation after 1000 h of storage under ambient conditions with 40% of relative humidity	Facilitates nucleation and growth of film, forms bulk heterostructure with reduced voids and defects, enhances charge transport and improves stability	24.90	[162]
2024	(TMA)_2_(FA)_n−1_Pb_n_I_3n+1_	Unencapsulated device maintained 88% of original efficiency at RT with relative humidity of 30% for a duration of 1080 h	Acts as the organic interlayer cation, form less low-n phase formation, better film quality and significantly improved electron mobility	16.56	[163]
2024	n-MoS_2_/p-MoS_2_	–	Functions as 2D absorber layer in vertically stacked Schottky and pn junction, enhances sunlight harvesting due to optimal electrical and optical properties	16.86	[164]
2024	(DF-BZA)_2_FA_3_Pb_4_I_13_ /Quasi-2D	Retained an average of 96% of original efficiency after 3000 h of storage in a N_2_-filled glove box	Act as absorber layer, improve film quality by enlarging grain size and increasing carrier lifetime	19.24	[165]
2024	TiO_2_/Cs_2_TiBr_6_/MoS_2_/PEDOT:PSS	–	Serve as HTL, offers high carrier mobility, better charge transport, reduce interface recombination and excellent chemical and thermal stability	19.29	[166]
2024	TC_6_Cl + Chlorine & Bromine Quasi-2D	Retained 972% of original efficiency after 1100 h of continuous light at 60% relative humidity at RT	Function as tailored hole transport materials, enhance energy-level alignment, improve hole extraction, passivate interface defects and reduce non-radiative recombination	21.07	[167]
2024	MeO-2PACz/ Cs(MAFA)Pb(IBr)/MPA/ BA_2_MA_n-1_Pb_n_I_3n+1_/PCBM/BGP	Retained 92% of its efficiency after 750 h storage in air around 2985 °C with 60% relative humidity	Integration of a thin passivating dipole layer eliminates energetic mismatch and electron extraction barrier Reduces surface defects, suppresses non-radiative recombination and improves interfacial charge extraction	21.53	[168]
2024	FA_0.6_MA_0.4_Sn_0.7_Pb_0.3_I_3_/C_60_-2NH_3_/C_60_/BCP/Cu	Maintained 90% of its efficiency after being stored under N_2_ atmosphere for 2400 h	Used as an interlayer, improves band alignment, enhances carrier mobility and suppresses non-radiative recombination at perovskite/C60 interface	21.64	[169]
2024	SnO_2_/MBene/perovskite/Spiro-OMeTAD	Retained 951% of PCE in air with 50% relative humidity at room temperature for 200 h	Forms a strong chemical bridge, enhancing charge transfer, aligning energy levels and passivating SnO_2_ surface defects	24.32	[110]
2024	(BDA)(MA)_n−_ 1Pb_n_I_3n+1_ /MXene	–	Bandgap tunable by varying layer number, overcome toxicity and stability issues, suppress pinholes and improve charge transport	24.60	[170]
2025	TiO_2_/MoSSe@MXene@TiO_2_/CH_3_NH_3_PbI_3_	Extended operation life and moisture resistance	Reduced work function of ETL for better interface alignment, facilitate charge extraction, suppress surface recombination and accelerate electron transport	13.50	[171]
2025	FTO/cp/mp-TiO_2_/MAPbl_3_:CN/C	–	Enhances crystallinity and facilitates charge transport via π-conjugated network	13.74	[172]
2025	ITO/Ti_3_CNT_x_/perovskite/Spiro-OMeTAD/Ag	Maintained 703% of its PCE after 600 h in the air	Optimized energy-level alignment, high conductivity, interacts with I^−^ ions to passivate defects and strong Pb–O bonds for enhanced stability	20.16	[173]
2025	FTO/TiO_2_/2D RP Perovskite/CNBThMA Spacer/Spiro-OMeTAD	–	Donor–acceptor CNBThMA spacer eliminates dielectric mismatch, optimizes energy-level formation, adjusts anisotropic charge transport and improves film quality	20.82	[174]
2025	SnO_2_/Perovskite:NbSe_2_-NP/MoO_3_/Ag	Maintained 81% of its initial PCE after 2400 h at 65% relative humidity and 25 °C	Strong coordination with Se^2−^/S^2−^ anions passivate defects, reduce trap density and extend charge carrier lifetime	23.03	[175]
2025	SnO_2_/perovskite:MBene/spiro/Au	Improved thermal stability and humidity, retains performance under long-term air exposure	Passivate uncoordinated Pb^2+^ improved vacancy formation energy and modulates crystallization via increased nucleation sites for improved film quality and reduced non-radiative recombination	24.22	[176]
2025	Planar p-i-n PSC/MoS_2_/FAPbI_3_/MoS_2_	Maintained 96% of original PCE after 1200 h at 85 °C at 85% relative humidity	Wafer-scale MoS_2_ buffers block ion migration, chemically stabilize FAPbI_3_ via Pb–S coordination and provide type-I band alignment to suppress minority-carrier losses	26.20	[32]

#### Optimizing Charge Transport for Enhanced Efficiency and Stability

**Fig. 6:**
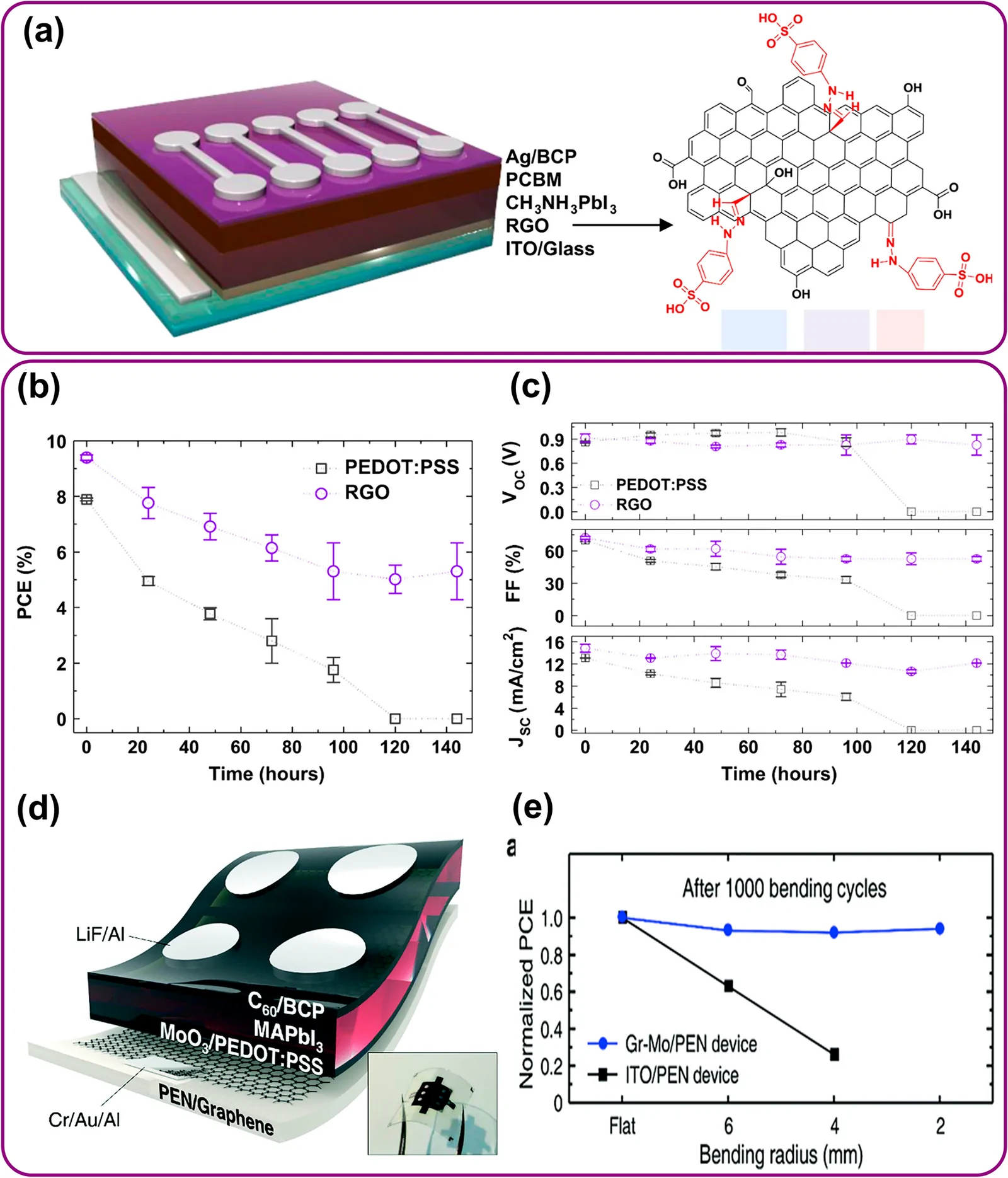
Optimizing charge transport for enhanced efficiency and stability. **a** Schematic diagram of RGO in MAPbl_3_ PSC for efficient hole extraction from PSC layer and wide bandgap of RGO minimize the energy loss during hole transport and contribute to higher V_OC_.** b**, **c** Long-term stability testing of PEDOT:PSS and RGO-based devices revealed that RGO exhibits superior chemical stability and effectively prevents perovskite layer degradation because of strong interfacial adhesion between RGO and the perovskite layer, **a**–**c** adapted with permission of ELSEVIER [177]. **d** Schematic diagram of graphene-based flexible PSC, where graphene delocalized π-electron facilitates efficient charge carrier transport and strong C–C bonds provide exceptional flexibility and resistance to bending stress. **e** Normalized PCE of ITO/PEN and Gr-Mo/PEN devices with varying bending radii; flat, 6, 4 and 2 mm after 1000 bending cycles. Gr-Mo/PEN devices showed remarkable flexibility and stability and retained 90% of their primary efficiency after 1000 bending cycles as compared to ITO which has inherent brittleness that led to crack formation and disrupted electrical conductivity in ITO/PEN devices, which result in a significant decline PCE during cyclic bending, **d**, **e** adapted with permission of ROYAL SOC CHEMISTRY [179]

Efficient charge carrier transport remains a critical challenge to achieve higher efficiency and facilitation of electron and hole extraction toward the external circuit, a suitable energy-level alignment and internal electric field need to be established among the device components. In one study, Yeo et al. presented the room temperature fabrication method for employing reduced graphene Oxide (RGO) as an innovative HTL in MAPbl_3_ perovskite solar cells [177]. High charge carrier mobility of RGO efficiently extracts the holes from the perovskite layer. The wide bandgap of RGO minimizes energy loss during hole transport which contributes to higher *V*_oc_, and high conductivity of RGO ensures the efficient hole collection at the CE which reduces the charge combination losses. The schematic diagram with the chemical composition of RGO is shown in Fig. 6a. RGO exhibits excellent chemical stability, preventing degradation of the perovskite layer and enhancing device lifetime. The strong interfacial adhesion between RGO and the perovskite layer improves device stability by preventing delamination. The device performance monitored w.r.t exposure time to an environment with approximately 50% relative humidity for checking the stability under ambient conditions according to *ISOS-D-1* protocol for degradation kinetics and mechanisms related to moisture-induced degradation processes. *ISOS-D-1* protocol is design to assess the stability of PV devices, particularly perovskite solar cells that focuses on dark storage condition aiming to evaluate the device tolerance to oxygen, moisture and other atmospheric components [178]. Solar cells-based PEDOT:PSS exhibited a rapid decline in PCE, resulting in complete PV performance loss within 120 h of exposure potentially due to a degradation mechanism attributed to erosion of the underlying ITO electrode induced by moisture ingress and ionic species migration within the layer. The photovoltaic parameters for PEDOT: PSS and RGO-based devices under ambient atmospheric conditions as a function of exposure time are shown in Fig. 6b, c. The acidic microenvironment caused by PEDOT:PSS induces perovskite decomposition through proton-mediated phase segregation; where proton separate the different components and phases within a material system potentially due to electrostatic interactions, hydrogen bonding and chemical reactions. The formation of volatile byproducts, such as methylamine and hydrogen iodide, with the presence of oxygen and moisture exacerbates these processes, leading to the formation of PbI_2_ and other degradation products. RGO-based device showed long-term stability due to its inherent properties that has a significantly slower degradation rate of perovskite layer. The low density of surface oxygen functionalities on RGO also creates an effective barrier against the ingress of oxygen and moisture that potentially reduces the degradation and device maintained 6% of their primary efficiency even after 140 h of exposure. In another study, Yoon et al. demonstrated a high-efficiency, flexible perovskite solar cell incorporating graphene as a transparent electrode. The delocalized π-electron system in graphene enables efficient charge carrier transport for effective charge collection and strong C–C bonds provide exceptional flexibility and resistance to bending stress. The device exhibited comparable PCE to a control device using a conventional ITO electrode, indicating graphene potential as an alternative to conventional electrodes [179]. The schematic of device structure is shown in Fig. 6d. The operational stability under repeated bending stress was evaluated by subjecting device to 1000 bending cycles at various curvature radii (*R*) of 2, 4 and 6 mm. Flexible perovskite cells utilizing ITO had significant performance degradation after repeated bending, and the device shows a 50% reduction in initial PCE after 1000 bending cycles at a radii of 4 mm. While the Gr-Mo/PEN devices exhibited exceptional stability under repeated bending, PCE remained at 90% of its initial value after 1000 bending cycles at both radii of 4 and 6 mm. The normalized PCE values of ITO/PEN and Gr-Mo/PEN devices after 1000 bending cycles are shown in Fig. 6e. The decrease in PCE for ITO/PEN devices under cyclic bending is primarily due to the intrinsic brittleness of ITO that induces crack formation within the film and disrupts its electrical conductivity and hinders the charge collection. While the graphene-based Gr-Mo/PEN device exhibits exceptional mechanical resilience due to its superior mechanical properties. The remarkable resistance of 2D material-based PSC to mechanical stress shows the ability to withstand repeated bending without substantial performance degradation making them promising candidates for flexible and foldable solar cell applications.

#### Role of 2D Materials in Improving Solar Cell Efficiency

2D materials such as graphene, MoS_2_ and TMDCs have emerged as promising interface modifiers in photovoltaic devices due to their unique optoelectronic properties. These materials enable precise band alignment tuning, enhance charge extraction, reduce carrier recombination and provide chemical passivation at critical heterojunctions in solar cells**.** By modifying energy-level offsets between the absorber, transport layers and electrodes, 2D materials directly influence key performance parameters such as open-circuit voltage (*V*_oc_), fill factor (FF) and PCE. This interface engineering strategy is particularly effective in perovskite and organic solar cells, where interfacial defects and non-radiative recombination often limit performance [180].

Najafi et al. [181] presented a comprehensive study on the use of graphene-related materials (GRMs), specifically MoS_2_ quantum dots (QDs) and functionalized reduced graphene oxide (f-RGO) as both HTLs and active-buffer layers (ABLs) in MAPbI_3_-based perovskite solar cells. The zero-dimensional MoS_2_ QDs, derived from exfoliated MoS_2_ flakes, exhibit enhanced hole extraction and electron-blocking capabilities due to quantum confinement and doping-induced intraband states. When hybridized with f-RGO via salinization chemistry, the resulting van der Waals heterostructure enables uniform film coverage, effectively sealing pinholes and improving interfacial contact. This strategy leverages the optoelectronic tunability and morphological compatibility of 2D materials to optimize device performance [182].

**Fig. 7:**
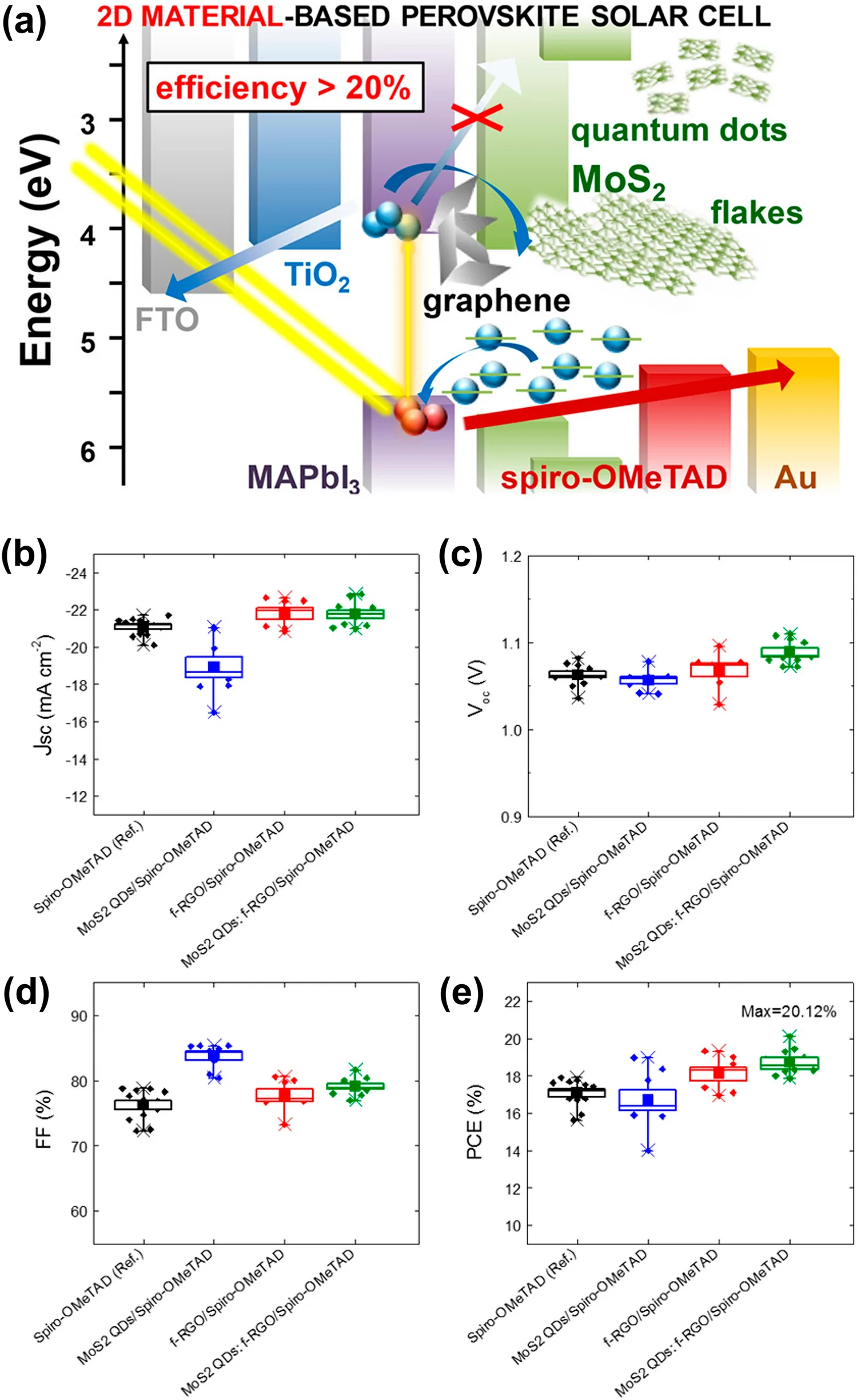
Role of 2D materials in improving the PCE of solar cells.** a** Energy band alignment in MAPbl_3_ PSC modified with 2D materials. **b–e** Photovoltaic performance of PSCs incorporating 2D material interlayers, showing **b** current density, **c** V_oc_, **d** FF and **e** PCE, adapted with permission from AMER CHEMICAL SOC [181]

Schematic Fig. 7a shows a schematic energy band diagram illustrating the band edge positions of the materials used in the assembled device. The conduction and valence band levels of MoS_2_ QDs and MoS_2_ flakes were experimentally determined using optical absorption spectroscopy (OAS) and ultraviolet photoelectron spectroscopy (UPS), while the band levels for other components, fluorine-doped tin oxide (FTO), TiO_2_, MAPbI_3_, spiro-OMeTAD and Au were adapted from previously published literature. The diagram emphasizes how incorporating 2D materials like MoS_2_ and graphene at the interface can improve energy-level alignment between the perovskite layer and the hole transport material, leading to more efficient charge extraction and suppressed recombination. Figure 7b–e summarizes the photovoltaic performance parameters of four different PSC configurations, including current density, *V*_oc_, FF and PCE: a control device with spiro-OMeTAD only, and three variants incorporating additional 2D additives MoS_2_ QDs, f-RGO and a hybrid MoS_2_ QDs:f-RGO interlayer. It is clearly observed that the 2D materials with optimal band gaps play an important role in improving the efficiency of the solar cells. Table 3 provides a detailed comparison of 2D materials in improving *V*_oc_, *J*_sc_, FF and PCE.

**Table 3:** A comparison of 2D materials in improving the photovoltaic features of solar cells

Material	*V*_oc_ (V)	*J*_sc_ (mA cm^−2^)	FF (%)	PCE (%)	References
WSe_2_/MoS_2_ (PbS QD)	0.42	–	43.00	7.65	[183]
BP:PCBM	0.81	16.00	0.64	8.30	[184]
MeBThMA-Pb	1.09	18.61	60.23	12.18	[174]
FTO/c-TiO_2_*/mp-*TiO_2_/ MAPbI_3_	0.95	23.50	48.50	13.50	[171]
h-BN/MoSe_2_	0.70	24.60	70.00	14.23	[185]
FTO/c-TiO_2_*/mp-*TiO_2_/MXene@TiO_2_/ MAPbI_3_	1.10	25.20	66.50	16.00	[171]
Ti_3_C_2_Tx-PSC	1.06	22.83	67.52	16.38	[173]
Spiro-OMeTAD	1.10	23.32	66.14	16.93	[186]
FTO/c-TiO_2_/*mp*-TiO_2_/MoS_2_/MXene@TiO_2_/ MAPbI_3_	1.15	26.30	67.70	17.40	[171]
Phosphorene/Au	1.08	23.32	0.71	17.85	[187]
FTO/c-TiO_2_/mp-TiO_2_/MoSSe/MXene@TiO_2_/ MAPbI_3_	1.19	27.50	68.80	18.50	[171]
MoS_2_ QDs/spiro-OMeTAD	1.06	20.98	83.02	18.98	[181]
f-RGO/spiro-OMeTAD	1.07	22.49	80.61	19.34	[181]
MoS_2_ QDs:f-RGO/spiro-OMeTAD	1.11	22.81	79.75	20.12	[181]
Ti_3_CNTx-PSC	1.13	24.16	73.96	20.16	[173]
CNBThMA-Pb	1.18	21.66	78.23	20.67	[174]
Perovskite/NbS_2_	1.13	23.95	82.50	22.32	[175]
Perovskite/NbSe_2_	1.14	23.95	84.40	23.03	[175]

### 2D Materials-Based Organic Solar Cells (OSCs)

#### Enhancing PCE Through Reduced Recombination by Employing 2D Materials as HTL and ETL

**Fig. 8:**
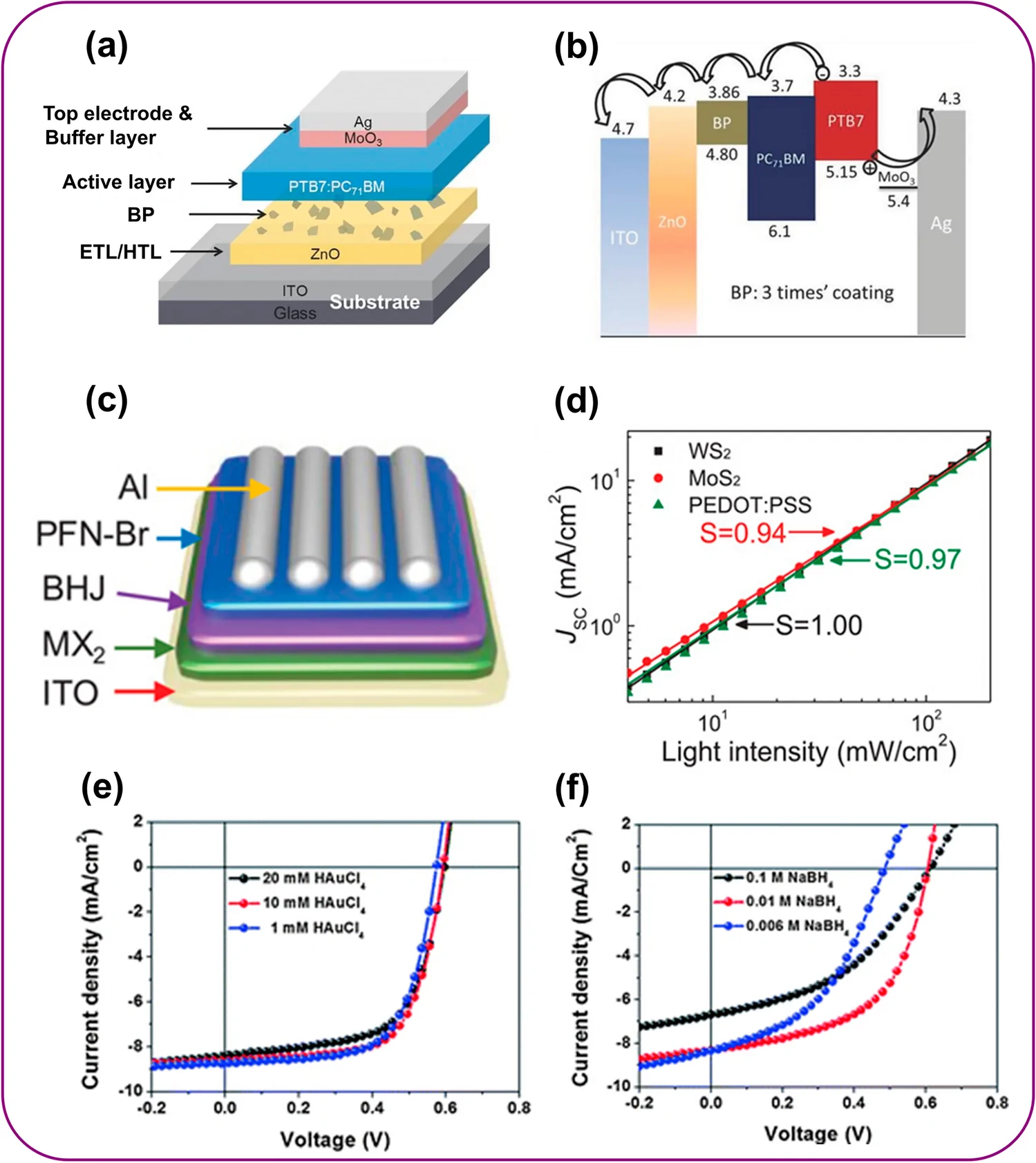
2D material as HTL and ETL for reduced recombination. **a** Schematic diagram of solution-processed BP as an ETL to create energy gradient between active layer and electrode for efficient flow of electrons. **b** Energy band structure; BP flakes create the cascaded band alignment to facilitate smoother electron transport and minimize energy loss, **a**, **b** adapted with permission of Wiley [35]. **c** Schematic diagram illustrates the use of liquid-exfoliated MX_2_ as a HTL for enhanced PCE by modifying the work function of MX_2_ to facilitate efficient hole transfer toward the electrode from the active layer. **d** Bimolecular recombination losses in different cell types with the influence of light intensity on the J_sc_ indicate WS_2_-based HTL demonstrated an S value of 1 shows a near-ideal scenario with minimal deviation from a linear relationship between J_sc_ and light intensity, (**c-d**) adapted with permission of Wiley [189]. **e–f** J-V curve of device with p- and n-doped MoS_2_ as HTL and ETL shows the effectiveness of work function engineering for optimized performance and enhanced charge transport within the device, (**e–f**) adapted with permission of ROYAL SOC CHEMISTRY [190]

OSCs have emerged as promising photovoltaic technologies due to their remarkable advancements in PCE. OSC offers several advantages that make them attractive for large-scale production. These include their lightweight nature, mechanical flexibility, partial transparency and compatibility with cost-effective roll-to-roll (R2R) processing techniques [20, 188]. The optimization of interlayer materials that are inserted between different components in OSC for improving the charge transport, modifying surface properties and enhancing device stability as well as electron donor and acceptors are key areas of research in organic photovoltaics to enhance device performance. In a recent study, Lin et al. explored the potential of solution-processed, ultrathin BP as an ETL. The introduction of BP with its unique bandgap creates a favorable energetic gradient between the electrode and the active layer that allows electrons to flow more readily from the perovskite layer toward the ETL [35]. The schematic of the device structure is shown in Fig. 8a. In some OPV materials, there may be a significant energy jump between donor and acceptor levels that hinder the efficient electron flow. BP flakes with a thickness of 10 nm are integrated into the OPV structure to create the cascaded band alignment that facilitates smoother electron transport, minimizing energy losses and improving device efficiency. The carefully chosen thickness of the BP flakes is critical for achieving the optimal energy band structure and a smoother energetic pathway of electrons for transport toward the collecting electrode that improved the average enhancement of 11% which resulted in increased PCE of 8.18%. The schematic representation of the energy band structure is shown in Fig. 8b. In another study, Yuan et al. introduced the potential of using liquid-exfoliated TMDCs, MoS_2_ and WS_2_ as a HTL in organic solar cells, achieving impressive PCE exceeding 17% [189]. The utilization of a solution-processing technique involves directly applying a suspension containing few-layered WS_2_ and MoS_2_ flakes onto the transparent ITO electrodes in the cell which simplifies device fabrication compared to traditional methods. The device structure is shown in Fig. 8c. The solution-processing approach results in modification for the work function of the WS_2_ layer without requiring additional treatments and facilitates the efficient holes transfer toward the electrode from the active layer. By varying the intensity of light on the solar cell, the generation rate can effectively be changed for electron–hole pairs and measuring the *J*_sc_ at different light intensities allows us to understand how efficiently these carriers are collected. The relationship between *J*_sc_ and *P*_light_ is shown in Eq. (1):

1 $$J_{{{\mathrm{sc}}}} \propto \left( {P_{{{\mathrm{light}}}} } \right)^{{\mathrm{S}}}$$

where the *S* represents the signature of recombination processes. If *S* = 1, it signifies a linear dependence of *J*_sc_ on *P*_light_ and suggests that all generated carriers are efficiently collected at the electrodes with minimal recombination losses. When *S* < 1, the *J*_sc_ increases slower than linearly while increasing light intensity, this deviation indicates the presence of bimolecular recombination that leads to a lower value of S. The influence of light intensity on the *J*_sc_ to quantify bimolecular recombination losses within the different cell types is shown in Fig. 8d. The OPV cell employing a WS_2_ as a HTL exhibits an S value of 1 that signifies a near-ideal scenario with minimal deviation from a linear relationship between the light intensity and *J*_sc_, while in MoS_2_ and PEDOT:PSS HTL cells they exhibit the values of 0.94 and 0.97, respectively, which indicates the presence of biomolecular recombination in these cells. This demonstrates that WS_2_ as HTL material offers a significant advantage in reducing bimolecular recombination compared to MoS_2_ and PEDOT: PSS which highlights the importance of 2D materials for optimizing the OPV cell performance. A study by Yun et al. explored the potential of work function engineered by MoS_2_ thin films for application in OSC. By strategically doping MoS_2_, the work function of the device was modified efficiently to extract hole for p-doped HTL or electron for n-doped ETL [190]. The improvement in PCE from 2.8% to 3.4% is because of the optimized energy-level alignment between the MoS_2_ layers and the adjacent organic materials in the OSC. The current–density (*J*–*V*) curve of devices with p- and n-doped MoS_2_-based HTL and ETL is shown in Fig. 8e, f. The consistent improvement in device performance across all configurations when MoS_2_ thin films were p- or n-doped compared to using undoped MoS_2_ as either a HTL or ETL suggests that the doping effectively engineered the MoS_2_ properties for better charge transport within the device. The improved PCE performance is potentially due to the modification of the work function of MoS_2_, aligning it more favorably with the energy levels of the adjacent materials that allow for smoother electron transfer to the n-doped MoS_2_ ETL from active layer, minimizing energy losses and boosting overall device efficiency [191]. Table 4 shows the PCE of 2D materials-based OSCs with their functional roles for enhanced charge extraction and improved energy-level alignment.

**Table 4:** Power conversion efficiency (PCE) of 2D materials-based OSCs with their functional roles for improved band alignment and interfacial layer

Device structure	Functionality	PCE (%)	References
Al/P_3_HT:PC_61_BM/MoO_3_-MoS_2_/FTO/Glass	Serve as HTL, after UVO treatment forms MoO_3_-MoS_2_ double layer which enhances hole extraction, blocks electrons and improved energy-level alignment	4.15	[192]
Al/P_3_HT:PC_61_BM(GQD)/MoS_2_/TFSA-GR	MoS_2_ layers improve charge extraction efficiency and interfacial resistance; TFSA-doped graphene acts as transparent conductive electrode and improves conductivity and energy-level alignment. GQD incorporation into active layer enhances light absorption, charge separation and improves flexibility and mechanical stability of device	4.23	[193]
Ag/p-MoS_2_/PBDTTT-C-T:PC_70_BM/n-MoS_2_/AgNW	Used as interfacial layers and composite transparent electrodes in AgNW which improves device fill factor and light absorption and enhances electrode conductivity and transmittance. Enables high-efficiency, large area and semi-transparency	6.39	[194]
Ag/MoO_3_/PTB_7_:PC_71_BM/PEIE/ITO/Glass	2D material work as ETL and sub-photosensitizer, which enhances charge separation and electron transfer. Improve light absorption, reduce electron decay time and enhance charge carrier dynamics	7.15	[195]
Al/PFN/ PTB_7_:PC_71_BM/MoS_2_@Au/ITO	Acts as HTL utilizing plasmonic effects from Au nanoparticles for increased light absorption and charge carrier generation to improve short-circuit current density	7.25	[196]
PFN/PTB_7_:PC_71_BM/PEDOT:PSS/MoS_2_	Serves as HTL, MoS_2_ modified with a surfactant to improve colloidal stability and solution processability for long-term storage and easy integration	7.26	[197]
GO/PTB_7_:PC_71_BM/LiF	Used as electron-blocking layer to replace PEDOT:PSS for enhancing thermal and humidity resistance and templating of active layer morphology, π stacked face-on microstructure	7.46	[198]
PTB_7_:PC_71_BM/e-MoO_3_	Serve as HTL, improves the hole transport and offers low-cost, scalable and efficient alternative to conventional vacuum-deposited MoO_x_ layers	7.54	[199]
Ag/MoO_3_/PBDTTT-C-T:PC_70_BM/ZnO/ITO	MoS_2_ nanosheets serve as interfacial layers and improve the charge transport, higher conductivity and transparency	7.62	[194]
Ag/MoO_3_/PTB_7_-Th:PC_71_BM/PEIE/ITO/Glass	Serves as ETL and auxiliary light harvester, facilitate efficient charge separation and accelerating electron mobility and improves optical absorption and shortens carrier lifetime	7.86	[195]
ITO/ZnO/PTB_7_:PC_71_BM/MoS_2_/Ag	Used as HTL, enhances the hole transport and energy-level alignment for room temperature, low-cost and scalable processing and alternative to vacuum-deposited MoO_x_ for large-area	8.10	[199]
PTB7:PC_71_BM/NbSe_2_	Acts as low trap density HTL, enhances the hole extraction and interfacial quality and effectively replaces MoO_3_ in OSCs	8.10	[200]
Ag/MoO_3_/PTB_7_:PC_71_BM:MoS_2_ NS/ZnO/ITO	Serve as dual-function additive in the ternary blend, enhance light harvesting by increasing optical absorption and improve charge transport by increasing carrier mobility and reduce trap state density	8.17	[201]
ZnO/BP/PTB7:PC_71_BM/MoO_3_	Used as ETL by forming cascaded band structure which facilitates electron transport, improve charge carrier extraction and reduce recombination	8.18	[35]
ITO/F-rGO/PTB_7_-Th:PC_71_BM/PFN/Al	Function as HTL, improve film formation and conductivity. Enhances the energy-level alignment due to higher work function fluorinated-reduced graphene oxide	8.60	[202]
CuSCN/AMQS/PTB7-PC_71_BM	Function as a passivating interlayer in bilayer hole extraction layer with CuSCN with reducing surface defects, suppress recombination and exciton quenching for enhanced charge extraction	8.80	[203]
ITO/MXene/PBDB-T:ITIC	Function as both HTL and ETL by modifying work function via surface treatment which enable selective contact formation and facilitates efficient tunable charge extraction	9.06	[204]
ITO/MoC:PEDOT:PSS/PTB-Th:PCBM/LiF	Serve as HTL and forms composite layer with PEDOT:PSS and enhances hole mobility, charge extraction and active layer morphology improved the device efficiency	9.24	[205]
ZnO/PTB_7_-Th:WSe_2_NFs:PC_71_BM/MoO_3_	Reduce exciton recombination, enhance charge transport, help in maintaining neat bulk heterojunction morphology and improve donor–acceptor interfaces and promote more balanced bipolar charge carrier mobility	9.24	[206]
ITO/PFN/PTB_7_:WSe_2_:PC_71_BM/MoO_3_/Ag	Function as third component within the active layer, promotes exciton generation and facilitates exciton dissociation at the WSe_2_-fullerene interface. Improve electron extraction and balance charge transport	9.30	[207]
RGO/ZnO:ITRGO/PTB_7_-Th:PC_71_BM/MoO_3_	Incorporation into hybrid ZnO-based cathode interlayers improve electrical conductivity, form interconnected nanostructures and aligns energy levels for enhanced charge extraction	9.49	[208]
MXene/PBDB-T:ITIC/PFN:Br	Function as HTL, improve the work function for efficient hole extraction and enhances electrical conductivity and optical transparency	10.53	[209]
α-In_2_Se_3_/PBDB-T:ITIC:PDINO	Incorporation with PEDOT:PSS forms a composite HTL, enhances conductivity by screening Coulombic interactions and improving PEDOT chain networking, offers suitable work function and enhanced optical transmittance	11.22	[210]
ZnO/PTB_7_-Th:IEICO-4F:BPNFs/MoO_3_	Function as morphology modifiers and enhances π-π stacking order and domain purity, reduce carrier resistance, suppress recombination and offers morphological stability by retarding phase mixing during device aging for stability	12.20	[211]
ZnO/PBDB-T:ITIC:Bi_2_OS_2_/MoO_3_	Function as heterogeneous nucleation in active layer, improve crystallization, charge transport and surface morphology for enhanced efficiency	12.31	[212]
PEDOT:PSS/Ti_3_C_2_TX/PM_6_:Y_6_/PFN:Br	Incorporation into PEDOT:PSS to form composite HTL for enhanced electrical conductivity and facilitates conformational changes in PEDOT, creates better charge transfer pathways	14.55	[213]
ZnO/PDINO-G/PM:Y/MoO_3_	Function as cathode interfacial material, for improved conductivity, lowers work function, reduce carriers recombination and enhances charge extraction	15.70	[214]
WS_2_/PBDB-T-2 F:Y/PFN:Br	Serve as HTL, WS_2_ enhances work function alignment, improves film uniformity, higher short-circuit current, reduced series resistance for enhanced performance	15.80	[215]

#### Enhancing Electron Collection by Reducing Work Function and Interface Barrier

**Fig. 9:**
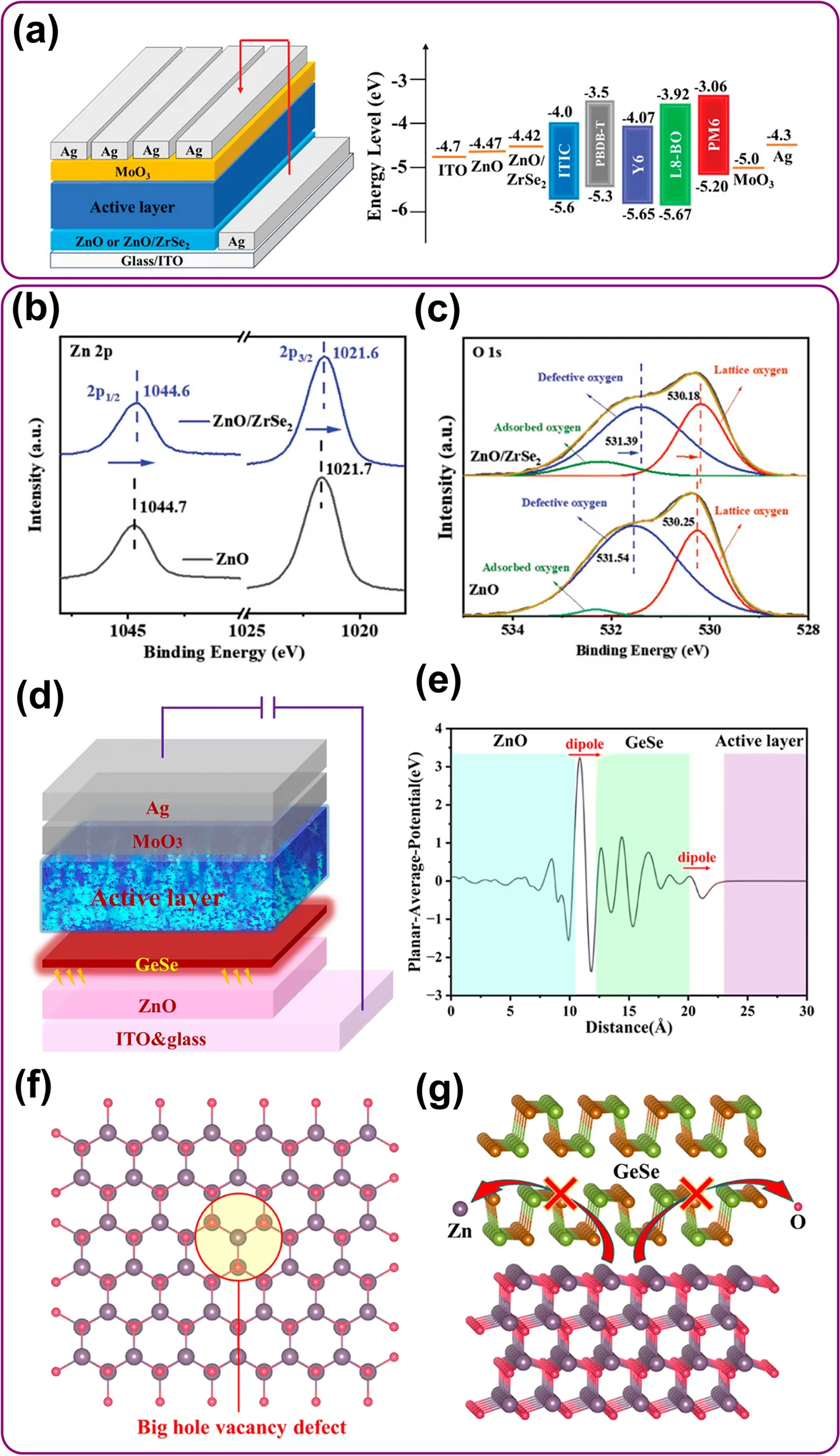
Reducing work function and interface barrier. **a** ZrSe_2_-modified ZnO ETL and energy band structure; for reducing work function and interface barrier between active layer and ETL to efficiently collect electrons. **b**, **c** XPS spectra of Zn 2*p* and O 1*s* with two prominent peaks at 1044.7 eV and 1021.7 eV corresponding to Zn 2*p*_1/2_ and Zn 2*p*_3/2_ show film shifted 0.1 eV toward lower energy from 1021.7 to 1021.6 eV and from 1044.7 to 1044.6 eV, respectively, because electron density increases around the Zn atoms lowers the work function of ZnO for efficient electrons injection into ETL for enhanced mobility of electrons. (a-c) adapted with permission of Wiley [216]. **d** Schematic structure of ZnO and GeSe composite ETL; the defect density on the ZnO surface is reduced by modification of ZnO with 2D GeSe. **e** Electron density change across the composite structure ZnO and GeSe. **f** Big hole vacancy defect form on polar surface of ZnO due to an imbalance of charges and creation of unstable sites. **g** 2D GeSe prevents the formation of ZnO defect vacancies to stabilize the crystal structure and reduce the defect formation for improved device efficiency, **d–f** adapted with permission of ROYAL SOC CHEMISTRY [119]

In a most recent study, Li et al. developed an efficient stable inverted OSC using a ZnO ETL modified with 2D ZrSe_2_. ZnO is commonly used as ETL due to its high electron mobility and good transparency [216]. However, the energy level of ZnO often does not align well with the active layer materials used in OSCs and hinder the electrons flow from the active layer to the ZnO ETL which leads to energy losses and reduced device efficiency. ZnO surface also has a high density of defects such as oxygen vacancies, dangling bonds and acts as recombination centers and reduce the overall cell performance. The mismatch between the active layer and ZnO leads to interfacial charge accumulation and device instability over time. The device structure of ZrSe_2_-modified ZnO ETL with energy level is shown in Fig. 9a. ZnO acquires electrons from ZrSe_2_ which causes the formation of interfacial dipoles that are regions of positive and negative charge at the interface between ZnO and ZrSe_2_. The formation of interfacial dipoles reduces the work function of ZnO which measure the energy required to extract an electron from the material, with lower work function facilitates the injection of electrons from the active layer into the ETL and leads to lower interface barrier between the ETL and active layer that enables more efficient electron collection and improve the overall cell performance. First-principles calculation (FPC) and XPS confirmed ZnO acquires electrons from ZrSe_2_ and form interfacial dipoles that reduce ZnO work function. XPS spectra of Zn 2*p* and O 1*s* are shown in Fig. 9b, c, where two prominent peaks observed at 1044.7 and 1021.7 eV correspond to Zn 2*p*_1/2_ and Zn 2*p*_3/2_, respectively. O 1*s* region have three primary peaks at 532.31, 531.54 and 530.25 eV that correspond to adsorbed oxygen, defect oxygen and lattice oxygen, respectively. Compared to pure ZnO film, the Zn 2*p*_1/2_ and Zn 2*p*_3/2_ peaks in the Zn 2*p* core level spectrum of ZnO/ZrSe_2_ film shifted 0.1 eV toward lower energy from 1044.7 to 1044.6 eV and 1021.7 to 1021.6 eV, respectively [217]. The increase in electron density around the Zn atoms minimizes the work function of ZnO and makes it easier for electrons to be injected into the ETL as well as enhances the mobility of electrons within the ZnO layer and improves the carrier transport in the OSC. The electron transfer and interfacial dipole formation potentially modify the interfacial properties between ZnO and the active layer and improve the charge transfer efficiency. In a recent study, Tan et al. introduced a novel ZnO&GeSe composite ETL by modifying ZnO with 2D GeSe which effectively reduced the defects density on ZnO surface and improved electron extraction efficiency in the devices [119]. GeSe can interact with ZnO to reduce the density of surface defects. The modification of ZnO with GeSe enhanced the electron extraction efficiency from the active layer to the ETL because of improved interfacial properties and reduced energy barriers. The schematic of device structure is shown in Fig. 9d. The interaction between ZnO and GeSe modifies the interfacial properties between the active layer and ETL, improves the charge transfer efficiency and reduces interfacial recombination. The incorporation of GeSe into ZnO alters the electronic structure of the ETL and creates a more favorable bandgap alignment with the active layer and reducing the energy barrier for electron injection. GeSe interacts with surface defects on ZnO, passivating them and reducing their ability to act as recombination centers. Differential charge density analysis shows the redistribution of electron density in a system. By comparing the electron density of the combined system, ZnO and GeSe to the electron densities of the individual components, we can identify regions of electron accumulation and depletion. The planar-averaged charge density difference in the ZnO/GeSe composite is shown in Fig. 9e. When ZnO and GeSe are brought together, some electrons from the surface of GeSe may transfer to the ZnO/GeSe interface. This electron transfer can be visualized as a region of electron accumulation at the interface and a region of electron depletion on the surface of GeSe [218]. The electrons transfer from GeSe to ZnO reduces the total energy of the system, as it seeks a configuration that minimizes energy. Differential charge density analysis shows the redistribution of electron density in a system. By comparing the electron density of the combined system, ZnO and GeSe to the electron densities of the individual components, we can identify regions of electron accumulation and depletion. The polar surface of ZnO is particularly susceptible to the formation of point defect vacancies that are missing ions from their regular lattice sites in a crystal due to imbalance of charges on the surface and create unstable sites; big hole vacancy defects as shown in Fig. 9f. Formation energy of point defect vacancy shows the influence of GeSe on ZnO defect formation; it is the energy required to create a defect in the crystal structure and lower formation energy indicates that a defect is more energetically favorable [219]. Point defect vacancies in ZnO significantly impact its electronic properties with single Zn point defects are often more metastable. They are energetically unstable but can exist for a relatively longer time, and the presence of a single Zn point defect can create a local distortion in the crystal lattice. Oxygen atoms near the defect site may experience an imbalance in electrostatic forces that drive these oxygen atoms to migrate to nearby face centered cubic (FCC) sites and create larger cavities on the ZnO surface. The schematic of GeSe which prevents the formation of ZnO defect vacancies is shown in Fig. 9g. The presence of cavities increases the surface roughness of ZnO which affects its optical and electronic properties. The defects and cavities also act as scattering centers for charge carriers which reduce the mobility of electrons. The modification of ZnO with GeSe potentially inhibits the formation of these defect vacancies. By interacting with ZnO surface, GeSe stabilizes the crystal structure and reduces the defect formation which leads to improved electronic properties and enhanced device performance [220].

Beyond ZnO, other wide-bandgap metal oxides such as tin dioxide (SnO_2_) have gained increasing attention as promising ETLs in inverted organic solar cells due to their high transparency, deep conduction band edge and low-temperature processability. SnO_2_ exhibits excellent chemical and thermal stability, and compared to ZnO, it possesses a lower density of oxygen vacancies, which reduces trap-assisted recombination and leads to enhanced electron extraction. Moreover, SnO_2_ has better energy-level alignment with many donor–acceptor systems, making it highly suitable for use in polymer-based OSCs [221]. For instance, SnO_2_ modified with surface ligands such as PEIE or doped with potassium ions (K⁺) has shown improved interface compatibility, lower work function and smoother film morphology, resulting in higher short-circuit current density (J_sc_) and fill factor (FF). These modifications reduce the interfacial barrier for electron transfer and suppress recombination losses.

In another approach, TiO_2_ nanoparticles and ZnSnO_3_ nanocrystals have also been explored as ETLs [222, 223]. TiO_2_ provides a high dielectric constant and suitable energy levels for many active layers, but often suffers from photocatalytic degradation under UV exposure [224]. To mitigate this, surface modifications (e.g., with graphene quantum dots or fullerene derivatives) have been employed to stabilize TiO_2_ and enhance charge transfer. ZnSnO_3_, a perovskite-type oxide, offers a deeper conduction band and better resistance to photocorrosion than ZnO, making it an emerging alternative for improving interfacial energetics and charge collection in OSCs.

Additionally, organic small molecules like bathocuproine (BCP) or perylene diimide derivatives (e.g., PDINO) are also frequently used either alone or in combination with metal oxides to improve interfacial properties [225, 226]. These layers can effectively reduce the cathode work function, enhance contact selectivity and passivate interfacial traps, resulting in better device stability and higher PCEs.

#### Enhancing Hole Collection via 2D Anode Modification Layers

While significant attention has been given to electron collection using 2D-modified cathodes, the anode side also plays a critical role in determining the overall efficiency of organic solar cells (OSCs) [227]. Several 2D materials, including GO, rGO, TMDCs (e.g., MoS_2_, WS_2_) and MXenes, have shown excellent potential as HTLs or anode interfacial modifiers due to their tunable work functions, good transparency and solution processability [103, 104, 180, 228].

The work function of 2D materials can be precisely tuned via chemical doping, surface functionalization, or plasma treatment to align well with the highest occupied molecular orbital (HOMO) level of the donor material, thus promoting efficient hole extraction and reducing energy barriers. For example, acid-treated GO or dopant-modified MoS₂ can enhance interfacial energy-level alignment with commonly used donors like PTB7 or PBDB-T [229]. Moreover, 2D HTLs can improve morphological compatibility, leading to better contact with the active layer and reduced interfacial recombination.

Beyond electronic benefits, these 2D layers also offer physical protection to the underlying electrode (e.g., ITO), reduce interfacial roughness and act as barriers against moisture and oxygen, enhancing device lifetime. For instance, MXene-based HTLs have demonstrated improved hole extraction, lowered series resistance and enhanced operational stability in flexible OSCs [109].

### 2D Materials-based Dye-Sensitized Solar Cells (DSSCs)

#### 2D Materials-Based Counter Electrode (CE) for Enhanced Efficiency and Stability

**Fig. 10:**
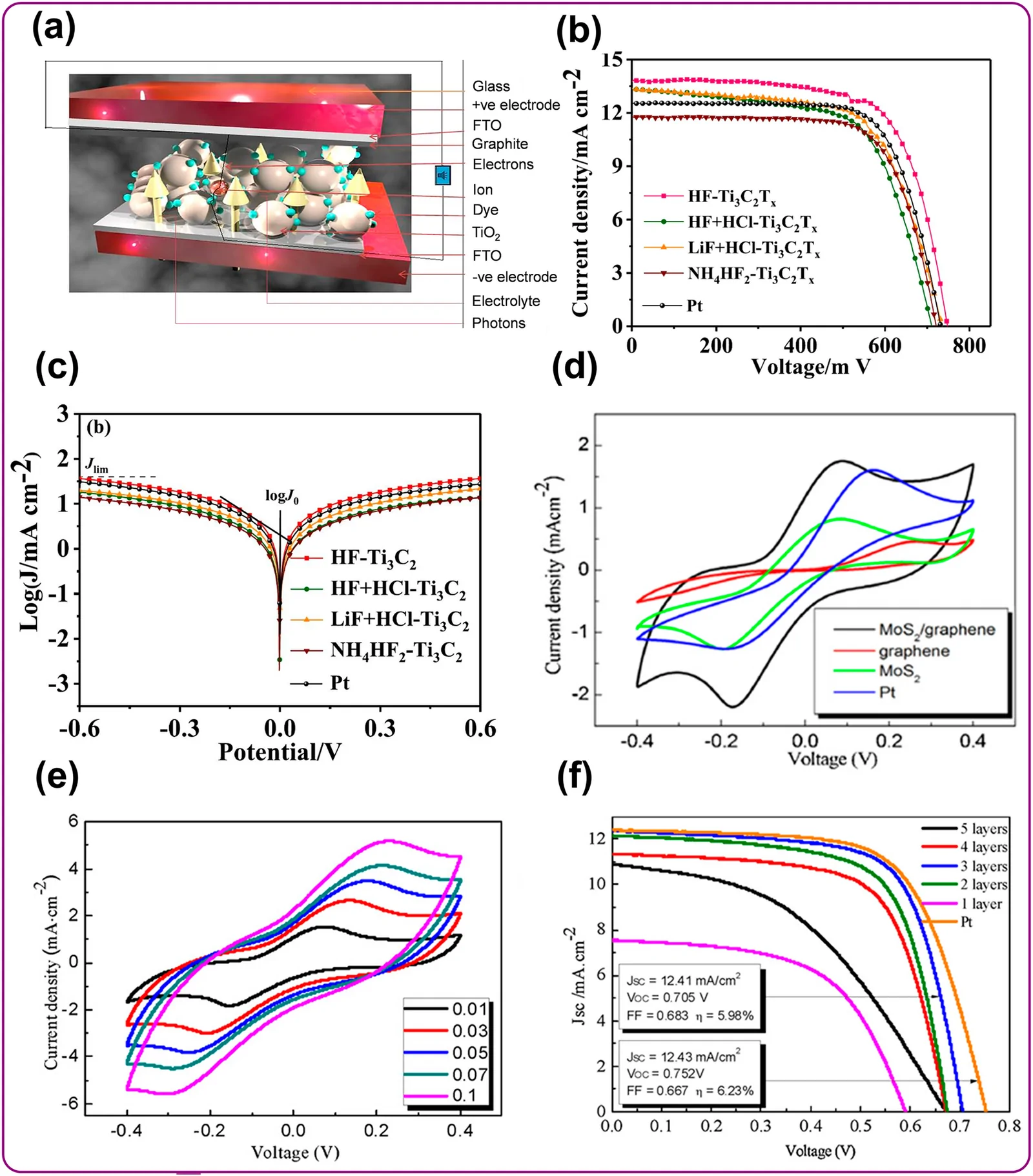
2D materials-based CE for enhanced efficiency and stability. **a** 3D schematic diagram of cross section of a DSSC with electrolyte and counter electrodes, adapted with permission of Nature Springer [238]. **b** *J*–*V* characteristics of four different Ti_3_C_2_ MXene CEs and Pt as CE. The highest *J*_sc_ for HF-etched Ti_3_C_2_ MXene CE is due to improved electrocatalytic activity for triiodide reduction and optimized pore structure with HF etching process creates a larger interfacial area and facilitates efficient mass transport of redox species. **c** Tafel polarization on symmetrical cells composed of identical pairs of the four Ti_3_C_2_ MXene and Pt counter electrodes in the electrolyte under dark conditions and exchange current density calculated from the intersection point of the anodic and cathodic Tafel regions, **b**, **c** adapted with permission of ELSEVIER [230]. **d** CV performed on the MoS_2_/graphene, graphene, Pt and MoS_2_ CE at a scan rate of 10 mV s^−1^ revealed an enhanced cathodic current density for the MoS_2_/graphene CE due to improved charge transfer kinetics and increased cathodic current. **e** CV for the MoS_2_/graphene composite at various scan rates. The cathodic and anodic peak currents shift systematically, as the scan rate increases and move toward more negative and positive potentials, respectively. **f** *J*–*V* characteristics of DSSC with Pt and MoS_2_/graphene CE of varying thicknesses. At higher scan rates, the double-layer charging current becomes more significant that influences the measured peak potentials, **d–f** adapted with permission of ELSEVIER [36]

DSSCs are emerging as promising photovoltaic technology due to cheap fabrication processes and sustainable energy sources with minimal environmental impact. These cells are composed of several key components working in tandem to convert sunlight into electricity. A photosensitizer, often dye, absorbs solar energy and injects electrons into a semiconductor that are then transported through the semiconductor to an external circuit. An electrolyte, a liquid or gel-like substance, contains redox species that regenerate the oxidized photosensitizer, and a counter electrode (CE) facilitates the return of electrons to the cell which catalyzes the reduction of the electrolyte. The entire assembly is supported by conductive glass to support the various layers of the DSSC. The schematic diagram showcasing a detailed cross section of a DSSC is shown in Fig. 10a. The performance of DSSCs is intrinsically linked to the material properties and molar extinction coefficient of the dye, which shows the material's ability to absorb light in the visible and infrared spectrum for efficient generation of photoinduced charge carriers within the photoanode.

In another study, Zhao et al. presented the synthesis of 2D-Ti_3_C_2_ MXene materials with varying surface terminations, particle sizes and sheet qualities through the selective etching of intermediate Al atoms from Ti_3_AlC_2_ for high-performance CE [230]. 2D-Ti_3_C_2_ MXene were explored as an alternative to conventional platinum (Pt) CE for triiodide reduction DSSCs. While Pt is an excellent catalyst for triiodide reduction in DSSCs because of its high stability and electrocatalytic, but high cost and scarcity limit its market competitiveness. Moreover, to achieve optimal performance, a relatively high loading of Pt is required which further increases the cost and potentially affects the device efficiency due to light blockage. Triiodide reduction occurs at the CE of a DSSC and involves the conversion of triiodide ions (${\mathrm{I}}_{3}^{-}$) into iodide ions (${\mathrm{I}}^{-}$) and crucial for regenerating the redox mediator in the electrolyte, which is essential for the continuous operation of the DSSCs. An efficient catalyst at the CE is required to facilitate this reaction and minimize energy losses. The etchant significantly influences the chemical properties of 2D-Ti_3_C_2_ MXenes that affects their catalytic activity toward triiodide reduction and PCE of DSSCs using these MXene-based CE varies with NH_4_HF_2_, LiF + HCl, HF + HCl and HF from 6.05%, 6.38%, 6.06%, to 7.15%, respectively. *J*–*V* characteristics with four different Ti_3_C_2_ MXene and Pt as CE are shown in Fig. 10b. The highest *J*_sc_ observed for HF-etched Ti_3_C_2_ MXene CE is due to enhanced electrocatalytic activity toward triiodide reduction and optimized pore structure, resulting from the HF etching process providing an expanded interfacial area and facilitating rapid mass transport of redox species and minimizing the electrode–electrolyte charge transfer resistance for improved electron transfer kinetics [231]. The increased surface area and reduced charge transfer resistance contribute to the superior performance of the HF-etched MXene counter electrode. Charge transfer kinetics, electrochemical impedance spectroscopy (EIS), interfacial properties of the electrolyte/electrode interface and Tafel polarization were investigated on symmetrical cells using identical pairs of the four Ti_3_C_2_ MXene and Pt counter electrodes in the electrolyte under dark conditions. The exchange J-V from the intersection point of the anodic and cathodic Tafel regions is shown in Fig. 10c, which shows the reaction reversibility and magnitude of charge transfer kinetics associated with the triiodide reduction process as shown in Eq. (2):

2 $${\mathrm{I}}_{3}^{ - } { } + { }2{\mathrm{e}}^{ - } { } \to { }3{\mathrm{I}}^{ - }$$

Tafel polarization evaluates the kinetics of an electrochemical reaction by measuring the relation between overpotential that shows electrode potential deviation from its equilibrium value and current density which measures the charge transfer resistance at the CE. EIS measures the interfacial properties of the electrochemical system that involves applying a small amplitude sinusoidal perturbation to the system and measuring the current response. It measures the resistance related to the electron transfer process occurring at the interface between the electrode/electrolyte and diffusion of ions within the electrolyte [232, 233]. In another study, Yue et al. presented a composite film composed of molybdenum disulfide (MoS_2_) and graphene flakes as a platinum-free CE for DSSCs [36]. The enhancement in electrocatalytic activity was observed when highly conductive graphene flakes were incorporated into the MoS_2_ film. The enhanced catalytic activity potentially due to multiple effects includes **(a) Increased active surface area:** the graphene 2D structure provides the large surface area for deposition of MoS_2_ nanoparticles and MoS_2_ edge sites that are highly active for electrocatalytic reaction increased surface area due to graphene, leading to higher number of active sites. **(b) Improved charge transfer:** graphene high conductivity efficiently transports electrons from MoS_2_ to external circuit accelerate the reaction kinetics and help to suppress charge recombination leading to higher overall efficiency. **(c) Enhanced mass transport:** the composite material exhibits the porous structure that promotes the diffusion of electrolyte ions to the electrode surface and increased mass transport enhances the rate of the electrocatalytic reaction. **(d) Electronic interaction:** the interaction between graphene and MoS_2_ potentially modifies the electronic structure of both materials leading to improved catalytic properties. **(e) Stabilization of active sites:** graphene helps to stabilize the active sites on MoS_2_, preventing degradation during the reaction [234]. Cyclic voltammetry (CV) curves obtained for MoS_2_/graphene, graphene, Pt and MoS_2_ CE at a scan rate of 10 mV s^−1^ are shown in Fig. 10d. CV analysis revealed an enhanced cathodic current density for the MoS_2_/graphene CE in comparison to graphene, Pt and MoS_2_. The higher cathodic current density in MoS_2_/graphene is due to improved charge transfer kinetics and increased cathodic current shows the rapid transfer of electrons from the electrolyte to the counter electrode surface, reducing the charge combination losses. The accelerated electron transfer rate can lead to a faster reduction of redox species in the electrolyte and improve the overall cell efficiency. Moreover, the lamellar structure of the MoS_2_/graphene composite may provide a favorable environment for the adsorption and reduction of redox species which lower the activation energy for the reaction, increase surface area and the synergistic effects between MoS₂ and graphene leads to a higher density of active sites for the electrocatalytic process [235]. CV of MoS_2_/graphene composite at different scan rates is shown in Fig. 10e. As the scan rate increases, the anodic and cathodic peak current densities exhibited a systematic, linear shift toward more negative and positive potentials, respectively. Due to increase in scan rate, the current flowing through the electrolyte increases which causes the voltage drop across the electrolyte, also known as Ohmic drop and distort the measured potential [36, 236]. The cathodic peak will shift toward more negative potential, while the anodic peak will shift toward more positive potential due to this Ohmic drop. The formation and relaxation of the electrical double layer at the electrode–electrolyte interface can also contribute to the peak shifts. At higher scan rates the double-layer charging current becomes more significant which can affect the measured peak potential. *J*–*V* characteristics of DSSC with MoS_2_/graphene and Pt CE of varying thickness are shown in Fig. 10f. MoS_2_/graphene composite film thickness was regulated by the application of adhesive plaster layers that exerts a substantial influence on the photoelectrochemical performance parameters of DSSCs utilizing MoS_2_/graphene CE including J_SC_, V_OC_ and fill factor (FF). The primary reason for increase V_OC_ and J_SC_ with thicker MoS_2_/graphene CE in DSSCs is a large catalytic surface area and properties toward the reduction of triiodide (${\mathrm{I}}_{3}^{-}$) ions regenerate the process of redox mediator in DSSCs. A thicker CE provide a larger surface area for catalytic reactions that lead to more efficient regeneration of the redox mediator and provide more pathways for electrons to travel from the photoanode to the external circuit and reduce resistance leads to higher *J*_sc_. A thicker CE can also potentially act as a barrier, reducing the recombination of holes and electrons at the interface between photoanode and the electrolyte which contribute to a higher *V*_oc_ [237]. Table 5 summarizes the performance of 2D material-based counter electrodes (CE) used in DSSCs.

**Table 5:** Performance of 2D materials-based CE for DSSCs

Material (CE)	FF (%)	*J*_sc_ (mA cm^−2^)	*V*_oc_ (V)	PCE (%)	References
2Co_0.85_Se/MoSe_2_/MoO_3_	67.1	13.8	0.768	7.10	[239]
Pt reference	69	14	0.75	7.2	[240]
NiSe	64	14.54	0.783	7.23	[241]
NiSe_2_	68	14.3	0.75	7.3	[240]
TaSe_2_ (solvothermal reaction)	64	15.81	0.73	7.32	[242]
MoSe_2_/graphene	60.41	17.12	0.71	7.34	[243]
NbSe_2_ (nanosheets)	63	15.04	0.77	7.34	[244]
FeSe_2_ (sphere-shaped)	69.8	14.6	0.724	7.38	[245]
CoSe_2_ (hydrothermal, 180	63.9	15.44	0.75	7.4	[246]
Pt reference	58	17.77	0.725	7.47	[247]
WSe_2_ (solvothermal reaction)	66	15.5	0.73	7.48	[242]
FeSe_2_ (nanosheets, under	60	17.49	0.718	7.53	[247]
MoSe_2_/PEDOT: PSS	67	15.97	0.7	7.58	[248]
FeSe (front irradiation)	61	17.1	0.733	7.64	[249]
FeSe_2_ microparticles (MPs)	66	15.63	0.745	7.68	[250]
Co_9_S_8_ (spin casting)	64	16.2	0.741	7.7	[251]
NbSe_2_ (nanosheets)	62	16.85	0.74	7.73	[252]
NbSe_2_/C	65	15.58	0.77	7.8	[244]
Pt reference	65	16.38	0.74	7.81	[248]
Pt reference	62.4	16.88	0.743	7.83	[246]
CoSe_2_/C–NR	67	15.98	0.73	7.83	[253]
Ni_0.85_Se (front irradiation)	63.6	16.67	0.74	7.85	[249]
Pt reference	70.2	15.13	0.741	7.87	[245]
Pt reference	69	15.88	0.72	7.9	[244]
Pt reference	67	16.84	0.7	7.91	[242]
Co_3_Se_4_	67	14.96	0.793	7.95	[241]
FeSe_2_ (3D power-like)	72.1	14.93	0.744	8	[245]
FeSe_2_ nanorods (NRs)	68	15.79	0.748	8.03	[250]
Pt reference	72.1	15.26	0.731	8.04	[254]
CoSe_2_ (hydrothermal, 140)	64.4	16.65	0.75	8.04	[246]
Pt reference	63	16.11	0.794	8.06	[255]
Pt reference	67	16.5	0.736	8.1	[251]
MoSe_2_/Mo (in situ selenization)	67	15.07	0.805	8.13	[255]
Pt reference	69	15.87	0.75	8.2	[250]
Pt@Ti reference	68	16.31	0.74	8.21	[248]
Pt reference	67	16.43	0.74	8.25	[253]
Co_0.85_Se (front irradiation)	66.8	16.74	0.742	8.3	[249]
Pt reference	69	15.33	0.791	8.3	[241]
Ni_0.85_Se	72	15.63	0.739	8.32	[256]
CoSe_2_ (hydrothermal, 160)	66.2	17.04	0.743	8.38	[246]
FeSe_2_ nanosheets (NSs)	70	16.14	0.744	8.39	[250]
CoSe_2_/C–NG	67	17.51	0.73	8.41	[253]
NiCo_2_S_4_ (spin casting)	66	17.4	0.743	8.5	[251]
MoSe_2_/PEDOT:PSS@Ti	69	16.41	0.75	8.51	[248]
Ni_0.67_Co_0.33_Se	69	15.89	0.784	8.59	[241]
Pt reference	73	16.03	0.738	8.64	[256]
Pt reference	68.3	17.19	0.74	8.68	[257]
MoS_2_/Mo (in situ sulfurization)	70.6	16.95	0.726	8.69	[257]
NiSe_2_ (hydrothermal reaction)	74.3	15.94	0.734	8.69	[254]
NiCo_2_S_4_/NiS (spin casting)	67	17.7	0.744	8.8	[251]
Ni_0.5_Co_0.5_Se	69	16.42	0.783	8.8	[241]
CoSe_2_/C–NCW	67	18.03	0.73	8.92	[253]
MoSe_2_/Mo (in situ sulfurization)	72.2	16.71	0.746	9.0	[257]
Ni_0.33_Co_0.67_Se	67	17.29	0.789	9.01	[241]
Ru_0.33_Se (front irradiation)	68.1	18.93	0.715	9.22	[249]
Co_0.85_Se	75	16.98	0.738	9.4	[256]
C–NCW/CoSe_2_ on carbon cloth	71	18.16	0.76	9.87	[253]
Gr/ MoSe_2_ (N_2_-doped)	70.07	19.73	0.724	10.01	[243]
C–NCW/CoSe_2_	71	18.86	0.78	10.46	[253]
Pt reference	73.22	19.93	0.723	10.55	[243]

#### 2D Materials-Based CE for Improved Electrocatalytic Activity

**Fig. 11:**
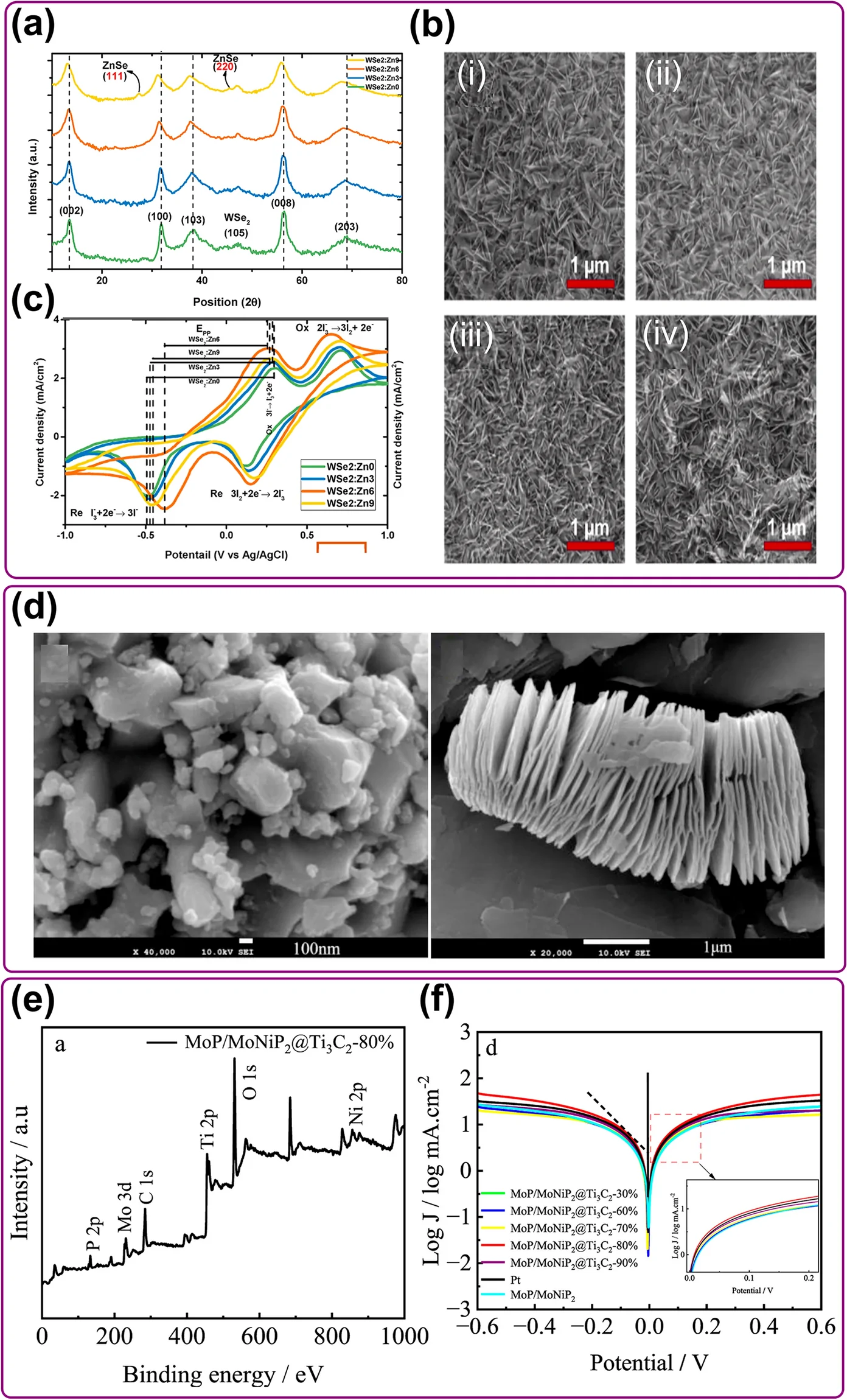
2D materials-based CE for improved electrocatalytic activity. **a** XRD spectra for WSe_2_:Zn_9_, WSe_2_:Zn_6_, WSe_2_:Zn_3_ and WSe_2_:Zn_0_ confirmed the formation of pure hexagonal WSe_2_ phase in prepared electrodes. **b** FESEM images of (i) WSe_2_:Zn0 (ii) WSe_2_:Zn_3_ (iii) WSe_2_:Zn6 and (IV) WSe_2_:Zn_9_ CE shows the electrodes layer composition with nanoflakes of WSe_2_ to enhance surface area for electrochemical reaction.** c** CV curves of different CE exhibit the anodic and cathodic peaks where WSe_2_:Zn6 CE shows elevated current density due to enhanced catalytic activity and presence of active sites facilitates electron transfer process, (a-c) adapted with permission of ELSEVIER [31]. **d** SEM images of MoNiP_2_/MoP nanoparticles with varying sizes. **e** XPS spectra of MoP/MoNiP_2_@Ti_3_C_2_-80% with the presence of Ti, C, Mo, O, Ni and P elements. **f** Tafel curve for different CE assesses the catalytic ability to reduce the I_3_^−^ and MoP/MoNiP_2_@Ti_3_C_2_-80% material provides active sites for the reduction of I_3_^−^ for enhancement in catalytic efficiency because the intercalation of MoP/MoNiP_2_ into Ti_3_C_2_ Mxene creates more open and porous structure for facilitation of I_3_.^−^ diffusion into electrode surface, **d–f** adapted with permission of ELSEVIER [260]

TMDCs have shown great potential for advanced PV devices because of exceptional electronics properties and comprises layers of transition metals bonded to chalcogen atoms are bound by weak vdW forces and exhibit high carrier mobility that reduces energy losses due to carrier recombination and scattering. In another study, Manal et al. proposed a novel CE for DSSCs utilizing crystalline tungsten diselenide (WSe_2_) doped with zinc (Zn) impurities. The CE is crucial in catalyzing the reduction of the oxidized redox mediator, typically iodide (I_3_^−^) to iodide (I^−^) for enhanced efficiency [31]. WSe_2_ high conductivity and catalytic activity toward the I_3_^−^/I^−^ is a promising material for CE and Zn doping has shown in enhancement of electrochemical and electrocatalytic properties. Zn doping increases the electrical conductivity of WSe_2_ leads to more efficient transfer of electron between the CE and electrolyte. It helps in faster reaction kinetics and lower overpotential due to the creation of active sites on the WSe_2_ surface that promote the reduction of I_3_^−^ to I^−^ with reduce charge recombination at the CE/electrolyte interface which is major loss mechanism in DSSCs. The crystalline structure and properties of the fabricated electrodes for WSe_2_:Zn_9_, WSe_2_:Zn_6_, WSe_2_:Zn_3_ and WSe_2_:Zn_0_ were characterized using X-ray diffraction (XRD) as shown in Fig. 11a. The observed peaks at 13.49°, 31.83°, 47.23°, 38.21°, 56.41° and 69.02° correspond to the (002), (100), (105), (103), (008) and (203) crystal planes of hexagonal WSe_2_ belonging to the P6_3_/mmc space group and align with the reference card number of 00-038-1388 that confirms the formation of prepared electrodes in pure hexagonal WSe_2_ phase [258]. While no additional phases were detected in the WSe_2_:Zn_6_, WSe_2_:Zn_3_ and WSe_2_:Zn_0_ samples that indicate the Zn doping did not introduce any significant impurities or secondary phases. WSe_2_:Zn_9_ sample exhibited two more peaks at 27.42° and 45.44° and the appearance of these peaks corresponds to the cubic ZnSe phase, suggesting that at higher Zn doping levels some of the WSe_2_ may undergo a phase transformation to ZnSe due to the increased concentration of Zn atoms, which can disrupt the WSe_2_ crystal structure and promote the formation of ZnSe. The surface morphology of the fabricated WSe_2_:Zn_9_, WSe_2_:Zn_6_, WSe_2_:Zn_3_ and WSe_2_:Zn_0_ electrodes examined using FESEM is shown in Fig. 11b. The FESEM image shows the electrode layers are composed of nanoflakes of WSe_2_ that enhance the surface area available for electrochemical reaction and improve performance. The presence of pores and crevices within the nanoflake structure facilitates the diffusion of electrolyte species and reduce mass transfer limitation contribute to faster electron regeneration at the CE/electrolyte interface. CE provides electrons to the electrolyte species and facilitates efficient regeneration and diffusion of N719 dyes. The structural properties facilitate both the rate of elecon regeneration in the electrolyte and the total current generated by the cell. The electrochemical and electrocatalytic properties of various fabricated CE evaluated using CV analysis are shown in Fig. 11c. The CV curves exhibit two distinct anodic (oxidation) and cathodic (reduction) peaks that correspond to redox reactions as mentioned in Eqs. (3) and (4), which interconvert the I_3_^–^ and I^–^ species.

3 $$ 2I_{2} + 2e^{ - }  \rightleftarrows 2I_{3}^{ - } $$

4 $$ I_{3}^{ - } + 2e^{ - } \rightleftarrows 3I^{ - } .$$

The higher *J*–*V* at this cathodic peak shows enhanced catalytic activity within the CE. WSe_2_:Zn_6_ CV shows the elevated current density for the cathodic peak which suggests a more rapid rate of I_3_^–^ species regeneration. The higher current density in WSe_2_:Zn6 is because of enhanced catalytic activity because the material possesses superior catalytic properties for this reaction and its surface structure, electronic properties and presence of specific active sites potentially facilitate the transfer of electrons process [259]. The large surface area of the catalyst also provides additional sites for the reaction to occur, and the addition of Zn might influence the morphology of the WSe_2_ which leads to higher density. The WSe_2_:Zn_6_ catalyst might also require a lower overpotential to initiate the reduction reaction compared to other materials where more of applied potential contributes to the reaction. In another study He et al. demonstrated CE based on MoP/MoNiP_2_@Ti_3_C_2_ composite with an efficiency of 10.01%. Transition metal phosphides (TMPs) have great potential as a promising research area for novel catalytic materials because of their structural similarities with transition metal nitrides [260]. The TMPs surface properties can be modified to enhance their catalytic activity by the addition of dopants or the creation of defects that improve the adsorption and desorption of species. A MoNiP_2_/MoP hybrid material was synthesized and intercalated into Ti_3_C_2_ MXene to create a "pillared effect". Intercalation causes the layer to expand and molecules of MoP/MoNiP_2_ act as pillars that prop up the Ti_3_C_2_ layers which increase the surface area of material that help in improve catalytic activity. SEM images of MoNiP_2_/MoP confirmed the presence of nanoparticles with varying sizes as shown in Fig. 11d. Due to pillared effect, the increased surface area provides more active sites for catalytic reaction and improves the efficiency of the catalyst. The SEM images show a range of nanoparticle sizes that indicate the heterogeneous nature of the material which influences the material catalytic and electrical conductivity. The layered structure observed in the SEM images is due to Mxene materials that arises from the intercalation of atoms or molecules between the layers of the material. The multiple layers suggest that the Ti_3_C_2_ MXene has a well-defined layered structure. XPS analysis of MoP/MoNiP_2_@Ti_3_C_2_-80% with the presence of Ti, C, Mo, Ni, O and P elements is shown in Fig. 11e that confirmed the successful synthesis of the composite [261]. Tafel polarization curves assess the kinetics of electrochemical reaction with material catalytic ability to reduce I_3_^–^ and plot the logarithm of the current density against the overpotential that is the difference between the equilibrium potential and applied potential of the reaction. The slope of the region is related to the transfer coefficient, which is a measure of symmetry for electrochemical reaction and at higher overpotential the current density reaches a plateau that indicates the reaction is limited by mass transfer. Tafel plots for different CE are shown in Fig. 11f where the composition of MoP/MoNiP_2_@Ti_3_C_2_-80% material potentially provides more active sites for the reduction of I_3_^–^ that helps in enhancement of catalytic efficiency. The intercalation of MoP/MoNiP_2_ into Ti_3_C_2_ MXene creates more open and porous structure that facilitate the diffusion of I_3_^–^ ions to the electrode surface. The combination of MoP/MoNiP_2_ and Ti_3_C_2_ may improve the electron transfer kinetics and allows for faster reduction of I_3_^–^ and contribute to a steeper Tafel curve [260, 262].

## Challenges and Limitations

This review highlights recent advancements in solar photovoltaics utilizing 2D materials. We discussed the 2D materials as promising candidates for next-generation photovoltaics applications and alternatives to fossil fuels. However, there are some challenges associated with 2D materials as well solar photovoltaic technologies that need to be addressed for improved performance, stability, efficiency and durability [263]. We have discussed these challenges and prospects as follows:

### Challenges with 2D Materials in Photovoltaics

2D materials due to their high carrier mobility, conductivity and optical transparency make them suitable candidates for next-generation photovoltaics. These materials have successfully been integrated as electrodes, HTL, ETL and active layer components in solar technology for improved light absorption and high efficiency. These materials can absorb light in visible and near-infrared regions and improved charge carrier extraction with high carrier mobility facilitates efficient carrier extraction from the active layer and reduces recombination losses [264, 265]. The optical properties of these materials can be tailored by controlling their thickness, composition and defect density which allows for optimization of specific applications [266].

Despite their promising properties, 2D materials face several challenges for their practical application in solar cells:

**(a) Limited light absorption:** Due to their thin structure, these materials have limited light absorption efficiency, especially in thicker regions of solar spectrum [39, 267].

**(b) Defect susceptibility:** The sharp edges and surfaces of 2D materials are more susceptible to defects, and these defects act as recombination centers and reduce overall cell efficiencies [268, 269].

**(c) Scalability:** The large-scale production of 2D materials remains a challenge that limits their commercial viability [270, 271].

To overcome these challenges, researchers are exploring hybrid structures for enhanced light absorption, defect engineering to minimize defect density and surface chemistry modifications for performance optimization. However, for real-world photovoltaic deployment, a deeper understanding of the fabrication and integration processes is essential.

### Challenges in Large-Scale Preparation and Integration

The commercial use of 2D material photovoltaics depends not only on their inherent optoelectronic benefits but also on whether it is possible to produce them on a wafer scale, control defects during exfoliation and transfer them without damaging them. Therefore, the scalability landscape is now dominated by three technical constraints: liquid-phase exfoliation (LPE), chemical vapor deposition (CVD) and post-growth transfer/lamination [272].

**(a) Chemical Vapor Deposition (CVD) Limitations and Uniformity**

CVD remains the only technique currently capable of producing homogeneous monolayers of MoS_2_, WS_2_ and graphene at centimeter to wafer-scale dimensions. However, conventional high-temperature (> 700 °C) growth often results in multi-nucleation sites, grain boundaries and thickness non-uniformities, which translate into shunt pathways in finished solar cells. Recent advances have addressed some of these issues: halogen-assisted metal–organic CVD (MOCVD) with in situ scavengers suppresses parasitic oxide formation [273], low-pressure and plasma-enhanced CVD processes enable growth at temperatures below 450 °C [274], and remote epitaxy or seed-free growth techniques have demonstrated single-crystal MoS_2_ across 8-inch wafers with less than 3% thickness variation [272].

Despite these significant improvements, further optimization of precursor flux, in situ passivation and thermal budget control remain essential to achieve defect-free junctions, especially for integration onto flexible polymer substrates.

**(b) Defect Control in Liquid-Phase Exfoliation (LPE)**

Although severe sonication and cavitation produce basal-plane vacancies, edge dangling bonds and solvent contamination that reduce carrier lifetime and photoluminescence, LPE offers the least expensive, ton-scale path to printable 2D inks [275]. More than 60% of these flaws can be repaired while maintaining flake yield by using controlled-shear or shear-mixing LPE, followed by surfactant exchange and mild thermal annealing (200–300 °C) [276]. Ionic-liquid co-solvents and machine learning-guided solvent selection have recently reduced the occurrence of Stone–Wales defects while maintaining 90% of the optical response in comparison to pristine flakes [277]. These techniques are making it possible to create conductive, low-recombination 2D dispersions for large-area solar module ink-jet printing and slot-die coating.

**(c) Transfer and lamination compatibility**

Most CVD films need to be incorporated into device stacks after being separated from their growing substrates. Wet chemical lifts that use polymer supports, like PMMA, leave behind wrinkles, residues and trapped bubbles that lower mobility and decrease Fermi levels [278]. Nowadays, residue-free MoS_2_ are produced via polymer-free dry-transfer techniques, such as selenium-mediated sacrificial layers, and are completely compatible with back-end-of-line temperatures (< 300 °C) [279]. Continuous lamination speeds of around 30 m min^−1^ on flexible PET or Cu-foil webs have been attained using roll-to-roll (R2R) electrostatic pick-and-place and laser-assisted release techniques [280]. Although alignment accuracy and contamination control are still active research topics, these advancements collectively suggest high-throughput assembly lines for 2D-based solar films.

### Challenges in Perovskite, Organic and DSSCs

Perovskite materials serve as an active layer in PSCs, offering excellent light absorption and tunable bandgaps. However, efficient charge carrier transport remains a critical challenge for achieving high stability and efficiency.

To address the challenges of charge carrier transport in PSC, researchers have primarily focused on two key strategies. **(a) Interface Engineering:** involves the optimization of the interfaces between the charge transport layers and the perovskite layer which is crucial for efficient charge collection and extraction [281, 282]. This involves the **(i) Energy-level alignment:** to ensure optimal energy-level offsets between the perovskite, ETL and HTL to facilitate efficient charge transfer, **(ii) Interface passivation:** to minimize the interfacial defects and trap states that can act as recombination centers and reduce charge carrier losses and **(iii) Interfacial charge extraction:** that includes the designing of interfacial materials with high charge carrier mobility and extraction capabilities to promote efficient charge collection [283]. **(b) Perovskite Composition and Morphology Engineering:** it involves the modifications of perovskite composition and microstructure that can significantly improve the charge carrier transport properties. The key approaches include **(i) Dimensionality control:** tuning the dimensionality of the perovskite structure (2D, 3D, or quasi-2D) can optimize the charge carrier diffusion length and mobility, **(ii) Crystal quality improvement:** it involves enhancing the crystal quality of perovskite film through optimized synthesis condition and post-treatment processes to reduce defect density and **(iii) Morphology control:** controlling the perovskite film morphology, i.e., orientation, grain, size and coverage, facilitates efficient carrier transport and reduces charge recombination [284, 285].

Organic solar cells (OSCs) have attracted significant attention due to their potential for flexible, low-cost and lightweight solar energy solutions. There are also some challenges associated that limit their efficiency and widespread adoption. One of the major challenges in OSCs is the recombination of photogenerated electrons and holes that reduces cell performance. These cells have lower electron mobility that hinders the efficient transport of electrons from the active layer to cathode. The interface barrier between the active layer and the ETL can create an energy barrier that hinders electrons injection and reduces cell efficiency. These challenges can be addressed through the following strategies; **(a) donor–acceptor blends with complementary properties.** To minimize recombination losses, morphology of active layer could be optimized using donor–accepter composite, and incorporating an interfacial layer to reduce charge trapping can help in achieving both the high performance and long-term stability of devices [286, 287]. **(b) Reduce work function.** Lowering the work function of ETL can facilitate the electron injection from the active layer, and improving the interfacial properties between the ETL and active layer potentially helps to reduce the interface barrier and enhance electron collection. This involves **(i) Doping.** Electron-rich dopants such as alkali metals or organic molecules can increase the electron density in the ETL, and co-doping of different dopants potentially leads to synergistic effects that optimize the work function for better performance [288]. **(ii) Surface treatments and interface modification.** Chemical or physical treatments of the ETL surface can alter its electronic properties and inserting a thin interfacial layer between the ETL and active layer can modify the electronic properties at the interface and lead to a reduction in work function. **(iii) Nano-structuring.** Engineering the morphology of the ETL at the nanoscale can influence its electronic properties. Porous and hierarchical structures can enhance electron transport and reduce the effective work function [289].

On the other hand, DSSCs face several challenges that hinder their widespread commercialization. One of the primary issues is **(a) long-term stability of the dye and** **electrolyte,** which significantly reduces the cell efficiency and lifespan due to degradation. While DSSCs have shown impressive efficiencies under ideal conditions, achieving comparable performance in real-world environments remains a challenge due to multiple factors such as humidity, temperature and light intensity, which affect the cell output [290, 291]. **(b) Electrolyte leakage** is another problem leading to degradation of the cell and potential safety hazards. Solid-state electrolytes offer a potential pathway to address the leakage issues, but these solid materials have lower efficiency and compromise cell performance [292]. The development of **(c) cost-effective and efficient counter electrode (CE)** is also a critical challenge for use on a commercial scale as an alternative to Pt. By addressing these challenges and implementing effective strategies, we can improve the efficiency and stability of solar photovoltaics and make them more competitive with traditional silicon-based solar cells for enhanced efficiency and stability [293].

## Conclusion

This review presents a comprehensive summary of the recent progress in photovoltaic technologies utilizing two-dimensional (2D) materials. The versatile roles of two-dimensional (2D) materials as hole transport layers (HTLs), electron transport layers (ETLs) and counter electrodes in perovskite solar cells (PSCs), organic solar cells (OSCs) and dye-sensitized solar cells (DSSCs) are emphasized. Their exceptional electronic, optical and structural properties enable enhanced charge transport, energy-level alignment, defect passivation and chemical stability are key factors for improving solar cell efficiency. In PSCs, 2D materials contribute to better perovskite crystallization, defect reduction and efficient charge extraction. In OSCs, 2D materials facilitate reduced recombination losses and improved interface engineering by tuning work functions and interfacial barriers. For DSSCs, 2D materials demonstrate promising electrocatalytic performance as cost-effective counter electrodes, contributing to improved stability and device performance. Despite these advancements, several challenges remain, including limited light absorption in some 2D materials, sensitivity to defects and difficulties in large-scale fabrication. Addressing these issues through interface engineering, doping strategies, surface modifications and scalable synthesis techniques is critical. Continued interdisciplinary research will be essential to fully realize the potential of 2D materials in next-generation, high-efficiency and stable photovoltaic technologies.

## Abbreviations

2D: Two-dimensional
BP: Black phosphorus
CIGS: Copper indium gallium selenide
CV: Cyclic voltammetry
DSSC: Dye-sensitized solar cells
EIS: Electrochemical impedance spectroscopy
EM: Electromagnetic
ETL: Electron transport layer
FESEM: Field emission scanning electron microscopy
FF: Fill factor
FPC: First-principles calculation
h-BN: Hexagonal boron nitride
HOMO: Highest occupied molecular orbital
HRTEM: High-resolution TEM
HTL: Hole transport layer
ITO: Indium tin oxide
LUMO: Lowest unoccupied molecular orbital
MHP: Metal halide perovskites
OFET: Organic field-effect transistors
OLED: Organic light-emitting diodes
OPV: Organic photovoltaic
OSC: Organic solar cells
PCE: Power conversion efficiency
PL: Photoluminescence
PSC: Perovskite solar cells
PV: Photovoltaic
QDSC: Quantum dot solar cells
RGO: Reduced graphene oxide
SAED: Selected area diffraction
TEM: Transmission electron microscopy
TMDC: Transition metal dichalcogenides
TMPs: Transition metal phosphides
XRD: X-ray diffraction
